# Binary Atomically Dispersed Metal‐Site Catalysts with Core−Shell Nanostructures for O_2_ and CO_2_ Reduction Reactions

**DOI:** 10.1002/smsc.202100046

**Published:** 2021-08-05

**Authors:** Xiaoxuan Yang, Maoyu Wang, Michael J. Zachman, Hua Zhou, Yanghua He, Shengwen Liu, Hong-Ying Zang, Zhenxing Feng, Gang Wu

**Affiliations:** ^1^ Key Laboratory of Polyoxometetalate Science of the Ministry of Education Faculty of Chemistry Northeast Normal University Changchun Jilin 130024 China; ^2^ Department of Chemical and Biological Engineering University at Buffalo The State University of New York Buffalo NY 14260 USA; ^3^ School of Chemical, Biological, and Environmental Engineering Oregon State University Corvallis OR 97331 USA; ^4^ Center for Nanophase Materials Sciences Oak Ridge National Laboratory Oak Ridge TN 37831 USA; ^5^ X-Ray Science Division Argonne National Laboratory Argonne IL 60439 USA

**Keywords:** atomic metal sites, CO_2_ reduction, core−shell structures, electrocatalysis, oxygen reduction

## Abstract

Engineering atomically dispersed metal site catalysts with controlled local coordination environments and 3D nanostructures effectively improves the catalytic performance for the oxygen reduction reaction (ORR) and the carbon dioxide reduction reaction (CO_2_RR), which are critical for clean energy conversion and chemical production. Herein, an innovative approach for preparing core−shell nanostructured catalysts with different single‐metal sites in the core and the shell, respectively, is developed. In particular, as the shell precursors, covalent organic polymers with a thin layered structure that is polymerized in situ and coated on a metal‐doped ZIF‐derived carbon core are used, followed by a controlled thermal activation. The selective combination and construction of different metal sites increase active site density in the surface layers, promote structural robustness, facilitate mass/charge transfer, and yield a possible synergy of active sites in the core and the shell. The p‐FeNC(shell)@CoNC(core), consisting of a polymerized FeTPPCl‐derived carbon layer (p‐FeNC) on a Co‐doped ZIF‐derived carbon (CoNC), exhibits remarkable ORR activity and stability in acidic media along with encouraging durability in H_2_–air fuel cells. Likewise, a p‐FeNC(shell)@NiNC(core) catalyst demonstrates outstanding CO_2_RR activity and stability. Hence, integrating two appropriate single‐metal sites in core and shell precursors, respectively, can modulate morphological and catalytic properties for a possible synergy toward different electrocatalysis processes.

## Graphical abstract

An effective synergistic catalysis strategy is developed to obtain atomically dispersed dual‐site catalysts with a core−shell architecture by allocating different isolated sites on the core and shell part via a step‐by‐step self‐assembly method, realizing different single‐site functionalizations. The unique method is versatile in regulating different catalytic activities by controlling the active sites of different building units.

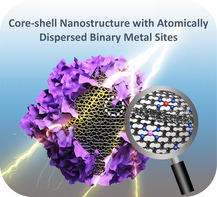

## 1 Introduction

Atomically dispersed and nitrogen‐coordinated single‐metal sites in carbon (M–N–C) have emerged as the most promising platinum‐group metal (PGM)‐free candidates in the field of electrocatalysis.^[^ 1, 2, 3, 4, 5, 6, 7, 8, 9 ^]^ The unique electronic structure and unsaturated coordination environments of the active sites in M–N–C catalysts have been proven highly active and selective for the oxygen reduction reaction (ORR) and CO_2_ reduction reaction (CO_2_RR).^[^ 10, 11, 12, 13, 14, 15 ^]^ However, due to the simplicity and limitation of single‐site centers, it is incompetent for addressing and regulating the trade‐off between activity and stability toward the ORR.^[^ ^16^ ^]^ For example, highly active single‐Fe‐site catalysts often deactivate rapidly, especially at the initial stage.^[^ ^17^ ^]^ Previous reports have shown that active site demetallation, carbon corrosion, and H_2_O_2_ attack could be primarily responsible for mitigating catalyst stability.^[^ 4, 17 ^]^

A catalyst containing two different single‐metal sites could expose increased atomic sites and yield a synergistic effect, providing a new approach to designing and engineering M–N–C catalysts with optimized morphologies, local geometric structures, and electronic environments.^[^ 18, 19 ^]^ For example, despite Fe–N–C catalysts exhibiting the most encouraging ORR activity among studied PGM‐free catalysts, a serious concern is the Fenton reactions between Fe^2+^ and H_2_O_2_, dramatically accelerating the ORR performance degradation.^[^ 5, 20, 21, 22, 23, 24 ^]^ In contrast, as a less active metal for the Fenton reaction, CoN_4_ sites can potentially address the severe stability issues, while delivering relatively low kinetic activity in challenging acidic media during the ORR.^[^ 25, 26, 27, 28, 29 ^]^

As another example, single Ni sites convey a high current density for the CO_2_RR toward CO production. However, they suffer from sluggish kinetics of the first proton‐coupled electron transfer (i.e., weak adsorption of *COOH), leading to a high overpotential.^[^ ^30^ ^]^ On the contrary, Fe−N_4_ sites have demonstrated low onset potentials for the CO_2_RR, whereas only maintained moderate activity in a very narrow potential window due to the strong adsorption of *CO on Fe−N sites,^[^ ^31^ ^]^ resulting in limited CO current density and poor stability.^[^ 32, 33, 34 ^]^ Therefore, integrating two types of single metal sites in a catalyst would be a promising and feasible strategy to push the performance and durability of PGM‐free catalysts beyond their current limitations. However, due to the competitive coordination with N ligands on the same catalyst, it remains a significant challenge to design and integrate atomically dispersed dual‐metal sites to catalyze electrochemical reactions effectively.

In this work, we developed a synergistic strategy to design atomically dispersed metal site catalysts with a core−shell nanostructure by allocating different single‐metal sites on the core and shell, respectively. The core−shell nanostructure can be tailored by controlling the inner‐ or outer‐layer components for a synergistic effect. In addition, the unique morphological characteristics may be beneficial for increasing the electrochemically accessible active site density and facilitating mass/charge transport during the electrochemical reactions. As the shell precursors, we used covalent organic polymers (COPs) with a thin layered structure obtained by in situ polymerization, which is coated on a metal‐doped ZIF‐8‐derived carbon (MNC), as the core, via a step‐by‐step self‐assembly method, followed by a controlled thermal activation. Carefully modulating the core size and shell thickness in the catalyst significantly increases the exposed density of active sites. Thus, it maximizes the catalytic performance of atomically dispersed M–N–C catalysts for the ORR and CO_2_RR.

## 2 Results and Discussion

### 2.1 Catalyst Synthesis, Morphology, and Structure

**Figure 1:**
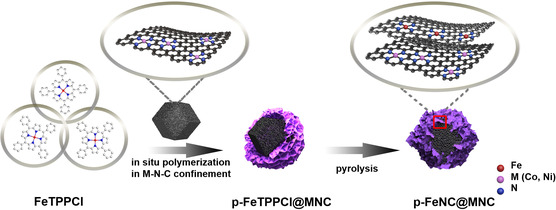
Schematic illustration of the preparation process for the core−shell nanostructured p‐FeNC(shell)@MNC(core) (M = Co, Ni, Fe) catalysts.

**Figure** **1** shows an effective method for synthesizing core−shell nanostructured catalysts with different atomically dispersed metal sites in the core and shell. First, as the core component, MNC (M = Co, Ni, or Fe) particles with a well‐developed porosity and high specific surface area were synthesized via two‐stage calcination according to our previous work.^[^ ^35^ ^]^ Next, they were exploited as templates for “in situ polymerization” to synthesize a porphyrin‐based shell on their surface. For example, tetraphenylporphyrin iron chloride (FeTPPCl) was chosen as a functional monomer and polymerized on the MNC core using aluminum chloride as a catalyst at 60 ^o^C. A controlled thermal activation was subsequently conducted to obtain core−shell nanostructured catalysts, denoted p‐FeNC@MNC, with atomically dispersed Fe sites in the shell and a different metal site (i.e., M) in the MNC core.

**Figure 2:**
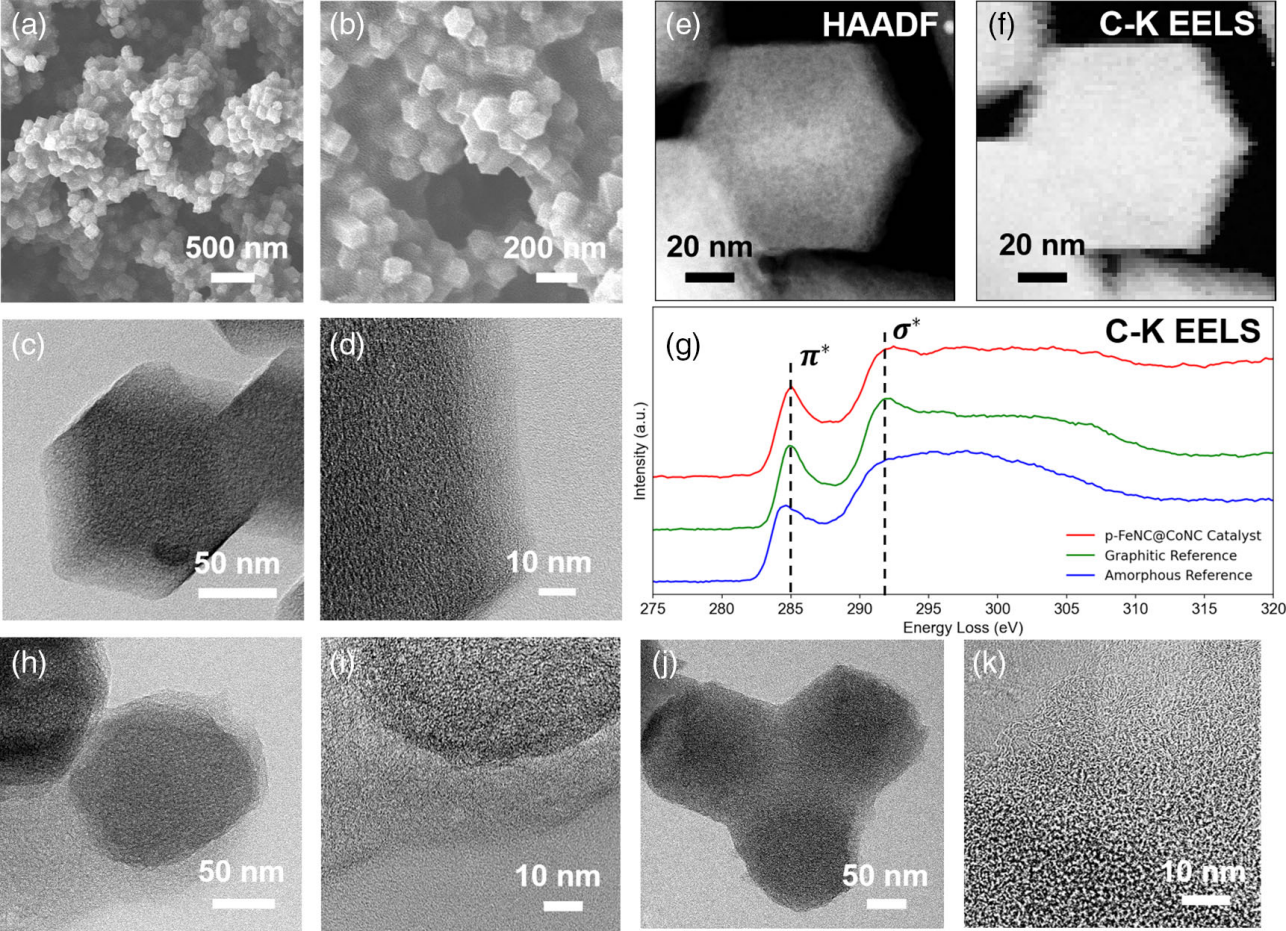
a,b) SEM, c) TEM, d) high resolution transmission electron microscopy (HRTEM), e) HAADF−STEM image, f) integrated carbon K‐edge EELS map, and g) the corresponding p‐FeNC@CoNC catalyst spectrum in addition to amorphous and graphitic carbon reference spectra. TEM and HRTEM images of h,i) the p‐FeNC@NiNC catalyst and j,k) p‐FeNC@FeNC catalyst.

The morphological and structural features of the prepared materials were studied with scanning electron microscopy (SEM) and transmission electron microscopy (TEM). For the p‐FeNC@CoNC sample, a unique network architecture with granular nodes was formed. The CoNC nanoparticles were randomly anchored and tightly coated with the p‐FeNC layer derived from the polymerized FeTPPCl (**Figure** **2**a,b and Figure S1a, S1b, Supporting Information). FeTPPCl was chosen as a novel shell precursor due to its superior properties, that is, well‐defined chemical composition, interconnectivity of building units, and versatility in molecular design.^[^ 36, 37 ^]^ In addition to FeN_4_ active sites in the shell layer, the MN_4_ sites in the core near the outer surface can participate in electrocatalytic reactions.^[^ ^38^ ^]^ On top of the core, a thin shell was formed with uniformly dispersed Fe active sites, which could fully be exposed and easily accessible to the reactants. As a comparison, the heat‐treated polymerized FeTPPCl without the CoNC core exhibited severe agglomeration, with uneven micrometer‐scale accumulations (Figure S2, Supporting Information). Therefore, as the support or template, CoNC nanoparticles greatly confined the in situ polymerization of FeTPPCl and effectively restricted the agglomeration during the polymerization and/or the subsequent carbonization process (Figure S3, Supporting Information). In addition, the FeTPPCl‐derived shell layer in the presence of the core part could generate increased surface areas and porosities, thus promoting single‐site dispersion and sufficient porosities for mass transport.

Our previous density functional theory (DFT) and experimental X‐ray absorption spectroscopy (XAS) studies^[^ 39, 40, 41 ^]^ indicated that, unlike the traditional CoN_4_ sites, the CoNC core contained substantial CoN_2+2_ sites.^[^ ^35^ ^]^ The single Co site is thermodynamically favorable for the 4e^−^ or 2+2e^−^ ORR pathway, which could be beneficial for stability improvement.^[^ 42, 43 ^]^ As a result, the FeN_4_ moieties with high activity in the shell could interact with CoN_4_ sites located in the core part, likely yielding isolated diatomic Co−Fe metal–nitrogen sites. Therefore, the tailored core−shell structures could modify catalyst morphologies for increasing exposed active site density. Compared with the original shape of the CoNC core, the coating does not cause a significant change in morphologies concerning the shapes and angular edges (Figure 2c,d and Figure S1c, S1d, Supporting Information). The interface between the core and shell parts is apparent. The shell layer is thin (≈10 nm), indicating that the CoNC core support can effectively disperse the p‐FeTPPCl shell.

Aberration‐corrected high‐angle annular dark‐field scanning transmission electron microscopy (HAADF−STEM) images and electron energy‐loss spectroscopy (EELS) were used further to reveal the detailed structure and morphology of the p‐FeNC@CoNC catalyst. The carbon particles in the catalyst are polyhedrons with an average size of around 130 nm. Unlike the amorphous‐dominated carbon structure in traditional Co−N−C catalysts,^[^ ^26^ ^]^ EELS of the carbon K‐edge revealed that the catalysts have a graphitic carbon lattice structure (Figure 2e–g and Figure S3, Supporting Information). The graphitic nature of the carbon could facilitate electron transport and enhance corrosion resistance during the electrocatalytic process.

In addition to the p‐FeNC@CoNC catalysts, other core−shell nanostructured catalysts with different core metal sites were synthesized using similar procedures. For the p‐FeNC@NiNC sample, a well‐coated core−shell structure with a medium‐thickness shell (≈20 nm) and rounded edges was produced (Figure 2h,i and Figure S4, Supporting Information). Unlike other catalysts, the p‐FeNC@FeNC sample displayed significantly different morphology, with the smoothest surface, the thickest shell, and the most obscure boundary between the core and the shell (Figure 2j,k and Figure S5, Supporting Information). Therefore, the morphologies and nanostructures of the catalyst shell are mainly dependent on the metal site of the carbon core. In particular, the Co‐doped carbon core possesses the lowest degrees of packing; the Ni‐doped carbon forms a medium‐thickness shell structure, and the polymerization degree is moderate; the Fe‐doped carbon core is the most easily coated, with the thickest shell and the tightest packing. Different metals in the cores likely affect the polymerization degree of the FeTPPCl shell precursors, thus leading to different core−shell structures and morphologies. In addition, in a core−shell structure, the porosity and graphitization degree of the carbon core, which are affected by the doping metal, significantly influence the thickness of the shell and the interaction between the core and shell.

**Figure 3:**
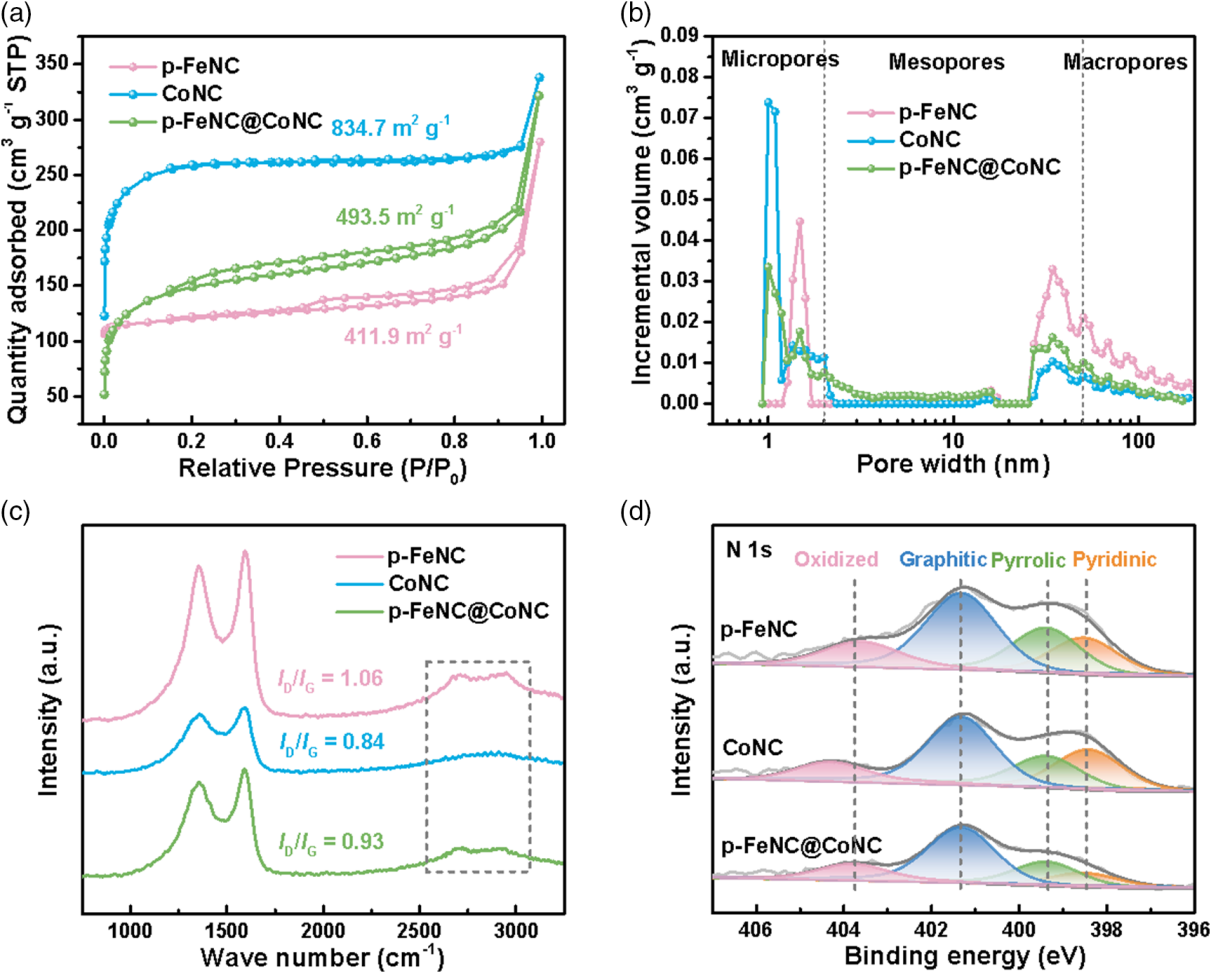
a) N_2_ adsorption‐desorption isotherms, b) pore size distribution, c) Raman, and d) XPS N 1*s* analysis for different samples.

N_2_ adsorption−desorption isotherms were conducted to determine the porous features of the different core−shell structured samples (**Figure** **3**a). The template‐free p‐FeNC sample exhibits a typical type I isotherm attributed to a dominant microporous property with a surface area of 411.9 m^2^ g^−1^. A remarkable increase in gas uptake at relatively high pressure (*P*/*P*_0_ > 0.90) reveals the existence of meso‐ or macropores, likely due to voids and interparticle porosity.^[^ ^44^ ^]^ Similarly, the CoNC core sample also shows a type I isotherm but with a more microporous structure and a higher specific surface area (834.7 m^2^ g^−1^). After assembling these materials into a core−shell structure, the p‐FeNC@CoNC possesses an H3‐type hysteresis loop with no major saturation adsorption platform, indicating an irregular pore structure. Its Brunnauer−Emmett−Teller (BET) surface area (493.5 m^2^ g^−1^) is also decreased relative to the CoNC core, likely due to the coating of the shell layer. Thus, compared with the individual CoNC core and p‐FeNC shell, the p‐FeNC@CoNC catalyst generated a hierarchical porous morphology with significant microporous/mesoporous structures (Figure 3b). As for desirable single‐metal‐site catalysts, sufficient micropores can accommodate active sites, meanwhile, mesopores can promote mass diffusion and transport.^[^ 45, 46, 47 ^]^

The carbon structures were further studied by Raman spectroscopy (Figure 3c). The prominent peaks at ≈1350 and 1590 cm^−1^ are designated as the D and G bands, respectively.^[^ ^48^ ^]^ Generally, the intensity ratios of *I*_D_/*I*_G_ are established to confirm the graphitization degree of carbon.^[^ ^49^ ^]^ The *I*_D_/*I*_G_ ratio value (0.93) of the p‐FeNC@CoNC sample is between the single‐component p‐FeNC (1.06) and CoNC (0.84). The CoNC core provides a higher degree of graphitization. Highly graphitized carbon enhanced electron transport and electrochemical stability but could accommodate less‐active metal sites. In contrast, the p‐FeNC shell has a relatively low degree of graphitization, exhibiting disordered and amorphous structures, which essentially provide defects to hold dense single‐metal sites despite its compromised stability. Therefore, integrating two different carbon structures in a core−shell nanocomposite could be desirable for an optimal activity and stability trade‐off.^[^ ^50^ ^]^ In addition, the peaks in the 2500−3500 cm^−1^ region suggest ordered structures in the p‐FeNC@CoNC sample with inherent characteristics of a 2D layered structure in the p‐FeNC shell.

X‐ray photoelectron spectroscopy (XPS) elucidated the composition and chemical state of the elements in the surface layers.^[^ ^51^ ^]^ Figure 3d and S6−S8, Supporting Information, show the N 1*s* and other XPS peaks. Tables S1−S3, Supporting Information, summarize the surface elemental composition of different catalysts. Compared with the single‐component shell or core, the N content in the core−shell nanostructures is slightly reduced due to an additional thermal treatment process (Table S1, Supporting Information). In each of the catalysts, there are four individual peaks, corresponding to the dominant pyridinic N (≈398.5 eV), pyrrolic N (≈399.4 eV), graphitic N (≈401.2 eV), and oxidized N (≈403.5 eV) (Figure 3d). For the p‐FeNC@CoNC catalyst, the orbital peaks of the survey spectrum present Co and Fe elements (Figure S8 and Table S1, Supporting Information) and indicate dominant oxidation states of Co and Fe single sites coordinated with N ligands.^[^ 52, 53 ^]^ The content of graphitic N (51.4%) was found to be increased compared with other individual components (Table S3, Supporting Information), which agrees with the Raman analysis. The graphitized carbon matrix helps enhance carbon corrosion resistance, which is thereby expected to promote catalyst stability.

**Figure 4:**
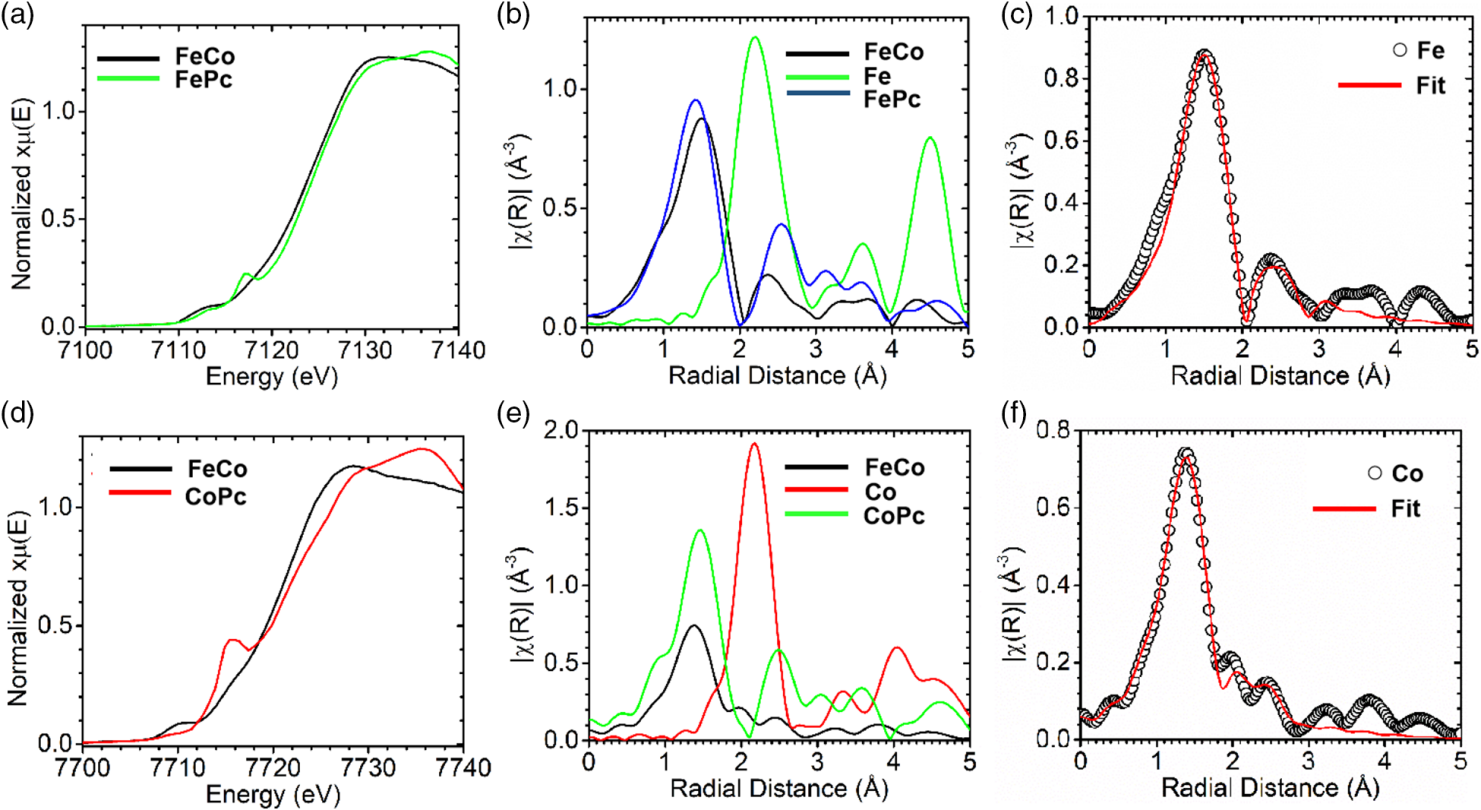
a−c) Fe and d−f) Co K‐edge XANES spectra, fit of the R‐space EXAFS, and the Fourier‐transform R‐space fitting for the sample p‐FeNC@CoNC (denoted as FeCo), respectively.

To further confirm the coordination environment of two different types of metal sites in the core−shell structured p‐FeNC@CoNC catalyst, XAS analysis and fitting were conducted (**Figure** **4**).^[^ 54, 55 ^]^ The Fe K‐edge X‐ray absorption near‐edge structure (XANES) is left of the FePc reference, which indicates that the oxidization state of Fe atoms is lower for the catalyst than for FePc (Figure 4a). Similarly, the Co K‐edge XANES confirms that the Co oxidization state in p‐FeNC@CoNC is approximately equal to CoPc (Figure 4d). The modeled extended X‐ray absorption fine structure (EXAFS) fitting (Figure 4b,e) reveals no metal—metal bonds. The Fourier‐transform EXAFS spectra in the R‐space of Fe and Co exhibit a primary peak around 1.5 Å, which is attributed to Fe—N and Co—N bonds, respectively. To further confirm FeN_4_ and CoN_4_ modification, the model‐based EXAFS fitting was conducted (Figure 4c,f). Compared with the standard FePc and CoPc fitting results (Figure S9 and Table S4, Supporting Information), both Fe and Co in the sample are coordinated with almost four nitrogen atoms. Therefore, the XAS fitting analysis verifies two different single‐Fe and single‐Co sites coordinated with four N atoms anchored in the shell and the core, respectively.

### 2.2 Oxygen Reduction Activity and Stability

**Figure 5:**
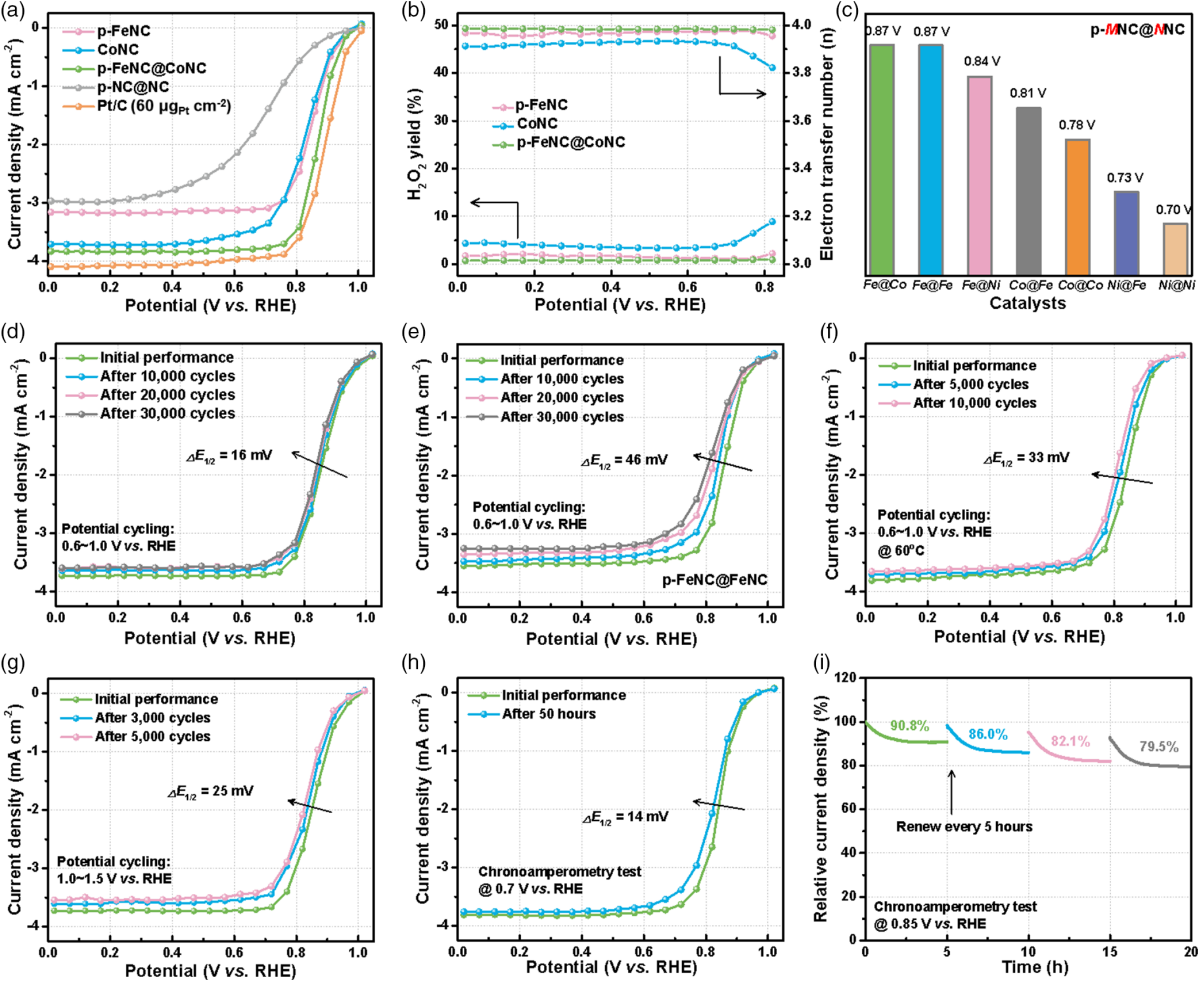
The ORR performance in 0.5 M H_2_SO_4_ solution. a) Steady‐state ORR polarization plots, and b) H_2_O_2_ yield and electron transfer number of different catalysts at 25 °C. c) Half‐wave potentials of shell@core structured catalysts with different metal elements. Potential cycling during low‐potential range (0.6−1.0 V vs RHE) for d) p‐FeNC@CoNC, e) p‐FeNC@FeNC at 25 °C, and f) p‐FeNC@CoNC at 60 °C in O_2_‐saturated 0.5 M H_2_SO_4_ solution. g) Potential cycling during the high‐potential range (1.0−1.5 V vs RHE) for the p‐FeNC@CoNC catalyst. h) Potentiostatic tests at 0.7 V versus RHE and i) 20 h chronoamperometry tests at constant potentials of 0.85 V versus RHE for the p‐FeNC@CoNC catalyst.

The ORR activity of the catalysts was evaluated using steady‐state staircase linear scanning voltammetry (SCV) measurement in O_2_‐saturated 0.5 m H_2_SO_4_ solution on a rotating disk electrode (RDE) (**Figure** **5**a). The metal‐free p‐NC@NC core−shell structured catalyst, as a control, exhibits poor activity, similar to N‐doped carbon catalysts.^[^ ^56^ ^]^ The ORR activity is effectively improved by introducing FeN_4_ species on a NC core, revealing FeN_4_ that sites at the surface are the dominantly active sites for the ORR (Figure S10, Supporting Information). The polymerization of FeTPPCl monomers could avoid metal agglomeration during carbonization, favorable for forming dense FeN_4_ active sites. The introduction of the NC support derived from ZIF‐8 nanocrystals could further expose more accessible active sites. After further introducing CoN_4_ into the core, the core−shell nanostructured p‐FeNC@CoNC catalyst only exhibits marginal enhancement of the ORR activity, compared with the FeNC@NC sample with dominant FeN_4_ sites in the shell. Importantly, these additional CoN_4_ sites in the core could remarkably enhance the catalytic stability, as verified by various accelerated stability tests (ASTs).

In particular, the p‐FeNC@CoNC catalyst delivers the best ORR catalytic activity, with an onset potential (*E*_onset_) of 0.98 V and a half‐wave potential (*E*_1/2_) of 0.87 V versus reversible hydrogen electrode (RHE) (0.6 mg cm^−2^ loading; 25 °C; 900 rpm in O_2_‐saturated 0.5 m H_2_SO_4_ solution), representing one of the highest RDE activities under similar experimental conditions for PGM‐free M−N−C catalysts (Table S5, Supporting Information). Furthermore, the core−shell nanostructured catalyst shows a lower H_2_O_2_ yield and higher selectivity toward the four‐electron ORR pathway than the single‐component catalysts (Figure 5b). Therefore, the deliberate selection of auxiliary polymerization and core supports is crucial for populating accessible active sites and promoting catalytic activity. We found multiple factors are critical to overall ORR activity during catalyst optimization, including metal content, shell‐to‐core thickness ratio, core sizes, heating temperatures, heating procedures, and core−shell structural effects. They have been carefully studied, and the relevant results are shown in Figure S11–S12, Supporting Information. We also found that the unique core−shell synthesis method is universal and effective in synthesizing other dual‐metal‐site catalysts (Figure 5c and S13–S14, Supporting Information).

Catalyst stability was further studied using extensive AST protocols. After 30 000 potential cycles (0.6–1.0 V vs RHE), the p‐FeNC@CoNC catalyst only lost 16 mV of *E*_1/2_ (Figure 5d). Unlike the continuous activity loss observed in the p‐FeNC@FeNC catalyst with a similar initial activity tested under the identical protocol (Figure 5e), the p‐FeNC@CoNC catalyst conveys remarkable stability after the initial degradation and better ORR activity after the AST. Previous DFT calculations have elucidated that, after the adsorption of molecular and atomic oxygen under an acidic environment, the Fe‐containing center became less stable.^[^ ^16^ ^]^ However, the structural stability of CoN_4_ sites is improved according to the calculations of the ion‐exchange energy landscapes on single‐Fe‐site and single‐Co‐site models.^[^ ^57^ ^]^ Furthermore, a single Co atom anchored in a carbon matrix would be hard to be demetallated, thereby mitigating the active sites’ loss and enhancing catalyst stability. Also, even under a harsher condition at a high temperature (60 °C), the catalyst can maintain relatively high activity (Δ*E*_1/2_ = 33 mV) after 10 000 cycles, indicating promising stability under similar fuel cell conditions (Figure 5f). The corrosion resistance of carbon in the p‐FeNC@CoNC catalyst was further assessed by cycling at a high potential range (1.0–1.5 V vs RHE) in a N_2_‐saturated 0.5 m H_2_SO_4_ solution. After 5000 cycles, the degradation of the catalyst is 25 mV loss in *E*_1/2_ (Figure 5g). The remarkable carbon corrosion resistance in the catalyst could be attributed to the high degree of graphitization in the core part.

Moreover, constant potential tests were conducted at potentials of 0.70 and 0.85 V versus RHE in an O_2_‐saturated 0.5 m H_2_SO_4_ solution. As a result, the p‐FeNC@CoNC catalyst conveys excellent stability due to an insignificant *E*_1/2_ (14 mV) loss at 0.70 V after 50 h (Figure 5h and Figure S15, Supporting Information). At 0.85 V, ORR polarization plots were recorded every 5 h, followed by different potential cycles (0−1.0 V vs RHE) (Figure 5i). During the stability test, the catalyst retains nearly 80% initial current density after 20 h. It presents a partially reversible activity recovery feature, likely due to removing the oxygen functional groups at the catalyst surface through the wide range of potential cycling. However, the overall degradation rates remain unchanged, likely resulting from inevitable demetallization. Overall, benefiting from the advantages of the two types of single‐metal sites in the core−shell structure, the p‐FeNC@CoNC catalyst provides excellent promise to balance activity and stability trade‐off.

The best‐performing p‐FeNC@CoNC catalyst was further evaluated as a cathode in membrane electrode assemblies (MEAs) (Figure S16, Supporting Information). Under 1.0 bar H_2_−air condition, the open‐circuit voltage (OCV) of the p‐FeNC@CoNC cathode (0.95 V) is higher than that of the p‐FeNC@FeNC cathode (0.90 V). As a result, the p‐FeNC@CoNC‐based MEA reached a critical current density of 46.4 mA cm^−2^ at 0.8 V, which is better than p‐FeNC@FeNC (27.0 mA cm^−2^). However, the maximum power density (*P*_max_) of the p‐FeNC@CoNC cathode is 0.30 W cm^−2^, lower than recent Fe/Co−N−C catalysts.^[^ 58, 59 ^]^ This is likely due to the dense surface of materials, along with the inevitable agglomeration of particles during the MEA preparation process, leading to uneven dispersion of the ionomers and reducing catalyst utilization, which would not be favorable for mass transfer.^[^ ^60^ ^]^ Given the auspicious intrinsic activity studied in an aqueous solution, further MEA design and fabrication optimization are required to achieve outstanding fuel cell performance. However, it should be noted that the MEA yielded a significant enhancement in power density after voltage cycling from 0.6 to 0.95 V for 1000 cycles under H_2_/air, which indicates that more internal active sites were exposed during voltage cycling, thereby gradually improving the performance. As a comparison, the p‐FeNC@FeNC cathode, containing only FeN_4_ in both shell and core, delivered the inevitable performance degradation in MEAs under the same conditions, further confirming that the alteration of Co sites in the core can result in considerable stability enhancement in challenging acidic media.

### 2.3 CO_2_ Reduction Activity

Besides the challenging ORR, we verified the effectiveness of the unique core−shell nanostructured catalysts toward the CO_2_RR through further regulating different active sites in the shell and core. In this case, Ni sites were successfully introduced into the core rather than Co. As a result, the core−shell structured p‐FeNC@NiNC catalyst and relevant control samples were further studied for the CO_2_RR using a three‐electrode H‐type cell in CO_2_‐saturated 0.5 m KHCO_3_ solution.

**Figure 6:**
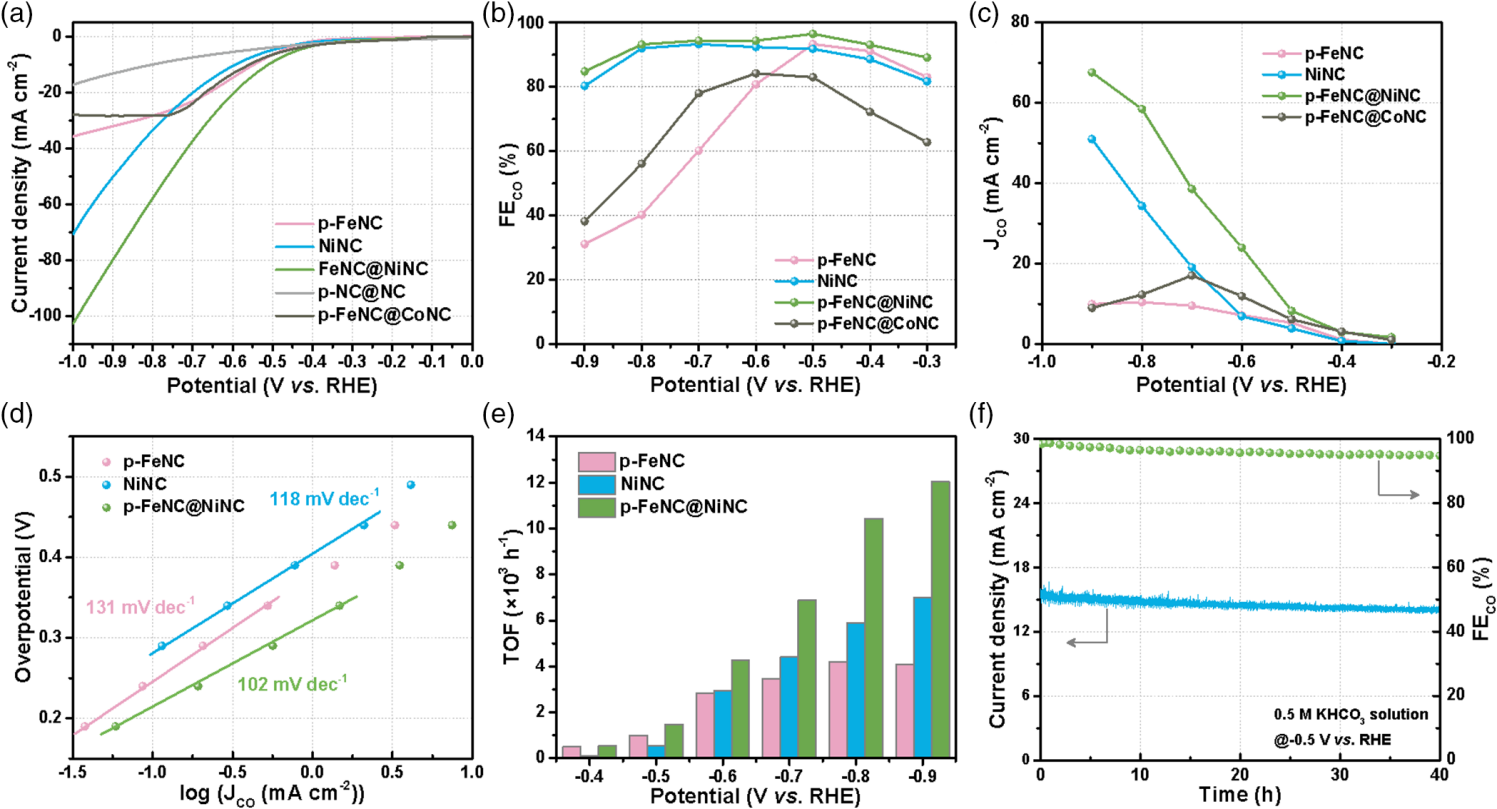
CO_2_ reduction performance in a CO_2_‐saturated 0.5 M KHCO_3_ solution. a) Linear sweep voltammetry (LSV) curves, b) FE_CO_, c) *J*_CO_, d) Tafel plots, e) TOF for different catalysts at various potentials, and f) stability test for the p‐FeNC@NiNC catalyst at −0.5 V versus RHE.

As shown in **Figure** **6**a, the p‐FeNC@NiNC catalyst with two metal sites shows the highest total current density (around 100 mA cm^−2^ at −1.0 V vs RHE) for the CO_2_RR, significantly exceeding that of catalysts with single‐metal sites in traditional Fe−N−C or Ni−N−C catalysts. CO was the primary product for the CO_2_RR catalyzed by the electrocatalysts, as analyzed via online gas chromatography. Faradaic efficiencies (FEs) vary with electrode potentials (Figure 6b). We calculated the partial CO production current densities (*J*_CO_) by combining the total current density and the corresponding FE_CO_ (Figure 6c). The p‐FeNC electrocatalyst exhibits an increased FE_CO_ from −0.3 to −0.5 V versus RHE, which could be attributed to the activation of CO_2_, and then a dramatic decrease at more negative potentials due to the CO_2_ transport limitation and kinetic competition between CO_2_ reduction and hydrogen evolution.^[^ 61, 62 ^]^ The previous DFT calculations revealed that FeN_4_ catalysts have a low onset potential for CO production due to the strong adsorption of the *COOH intermediate on the FeN_4_ site for activating CO_2_. However, the strong *CO binding energy could also suppress the desorption of CO from the active sites under high overpotentials, leading to a dramatic decrease in both CO production rates and selectivity.^[^ 63, 64, 65 ^]^ In contrast, due to the unique reaction kinetics, the adsorption of *COOH on the NiN_4_ site is thermodynamically more favorable than H* at high overpotentials, resulting in high FEs toward the CO_2_RR. Also, the *CO binding on NiN_4_ is relatively weak, which inhibits the further CO conversion, thereby producing a high current density for CO production. However, NiN_4_ sites suffer from sluggish kinetics of the first proton‐coupled electron transfer.^[^ ^66^ ^]^ As a result, the FE_CO_ on the NiNC catalyst is not prominent in the low‐overpotential region. The FE_CO_ increased with a continuously elevated overpotential, and reached a maximum value at a potential of −0.8 V versus RHE. Interestingly, the p‐FeNC@NiNC catalyst combines the advantages of the two active sites, that is, simultaneously maintaining excellent FEs in both low‐ and high‐overpotential regions. Specifically, it exhibits high CO selectivity with a maximum FE of *≈*97% at −0.5 V versus RHE and also dramatically outperforms the catalysts with individual metal sites across the entire potential window from −0.3 to −1.0 V versus RHE, suggesting that Fe/Ni sites synergistically inhibit the hydrogen evolution reaction (HER) at high overpotentials. The p‐FeNC@NiNC catalyst demonstrated competitive CO formation over a wide potential range and outperformed most reported CO_2_ reduction catalysts (Table S6, Supporting Information). For example, at −0.8 V versus RHE, the *J*_CO_ of p‐FeNC@NiNC reaches up to 58.5 mA cm^−2^, representing an improvement factor of 5.6 and 1.7 times compared with the p‐FeNC and the NiNC samples, respectively. It should be noted that the p‐FeNC@CoNC catalyst with FeN_4_ and CoN_4_ sites exhibits an insufficient activity for the CO_2_RR, further verifying the essential roles of the combination of FeN_4_ and NiN_4_ species in the shell and core, respectively.

To reveal the reaction kinetics, the corresponding Tafel plots were analyzed (Figure 6d) for the CO_2_RR. The p‐FeNC@NiNC catalyst exhibits the lowest Tafel slope of 102 mV dec^−1^, revealing that CO_2_ reduction proceeds through a mechanism where the first electron transfer is the rate‐determining step.^[^ ^67^ ^]^ Also, it shows a smaller Tafel slope than p‐FeNC (131 mV dec^−1^) and NiNC (118 mV dec^−1^), evidencing the enhancement effect of dual‐Fe/Ni sites. The intrinsic activity is further evaluated by calculating the turnover frequency (TOF) (Figure 6e). The calculated TOFs at −0.9 V versus RHE for the p‐FeNC, NiNC, and p‐FeNC@NiNC catalysts are 4071, 6980, and 12 058 h^−1^, respectively, representing the superiority of the core−shell structure with dual sites in enhancing the CO_2_RR activity. The TOF value of p‐FeNC@NiNC is comparable with the best catalysts for electrochemical CO_2_‐to‐CO conversion (Table S6, Supporting Information).

For designing effective core−shell electrocatalysts for the CO_2_RR, favorable thermal activation, particle size, feed ratio, and Ni content in the precursor are required. For a more intuitive comparison, Figure S17, Supporting Information, shows the FE_CO_ and corresponding *J*_CO_ for the p‐FeNC@NiNC catalyst, presenting a correlation between the single‐factor variable and CO_2_RR performance. Furthermore, the p‐FeNC@NiNC catalyst maintains 91% of the initial current density (around 15.3 mA cm^−2^) with 95.5% of the initial FE_CO_ after a 40 h stability test at −0.5 V versus RHE (Figure 6f), demonstrating outstanding electrochemical durability. In contrast, the p‐FeNC@FeNC catalyst containing individual FeN_4_ sites delivers poor stability with ≈37.7% *J*_CO_ loss after 12 h of the potentiostatic test (Figure S18, Supporting Information). Thus, further engineering two types of single‐metal sites in the core and shell could present great promise to improve the catalyst stability for the CO_2_RR significantly.

## 3 Conclusion

In summary, we developed an effective approach to designing and synthesizing atomically dispersed dual‐metal‐site electrocatalysts with core−shell structures for the ORR and the CO_2_RR. The two different MN_4_ moieties with sophisticated functionalities were separately distributed in the shell and core regions, avoiding coordination competition due to limited N ligands. The thin shell derived from COPs (e.g., in situ‐polymerized FeTPPCl) ensures that highly active FeN_4_ sites are uniformly distributed in the surface layer with favorable mass and charge transports. As the support or template, ZIF‐derived MNC nanoparticles can effectively disperse the in situ‐polymerized FeTPPCl, suppress the agglomeration of monomers, and promote the maximized exposure of FeN_4_ active sites in the shell. Amorphous carbon in the shell essentially helps provide sufficient defects for hosting dense metal sites on a core with a high degree of graphitization, simultaneously achieving a high density of sites and enhanced stability. In addition, the structure and morphology of the core, which is related to the type of incorporated single‐metal sites (e.g., Fe, Co, or Ni), greatly affect the thickness of the shell and the interactions between the core and shell, therefore governing overall catalytic activity and stability. The unique integration of the shell and core containing various metal sites provides a versatile route to producing high‐performing electrocatalysts for different reactions concerning selectivity, activity, and stability.

In particular, a variety of such core−shell structured catalysts were carefully examined for two critical electrocatalysis processes: O_2_ and CO_2_ reductions. Among others, the p‐FeNC(shell)@CoNC(core) catalyst exhibited compelling catalytic properties for the ORR and H_2_−air fuel cell performance. Meanwhile, the p‐FeNC(shell)@NiNC(core) catalyst yielded excellent CO_2_RR activity and stability for CO production. Although deeper understanding is needed concerning the possible synergy between single‐metal sites in the shell, core, and interfaces, this work opens up enormous possibilities for designing advanced atomically dispersed single‐metal‐site electrocatalysts for critical energy‐conversion electrochemical reactions.

## 4 Experimental Section

4.1

4.1.1

##### Synthesis of CoNC Template

Typically, F127 (1 g) was dissolved in methanol (25 mL) and then mixed thoroughly with methanolic solution (25 mL) of zinc nitrate hexahydrate (2.68 g, 9 mmol) and cobalt (II) nitrate hexahydrate (1.16 mg, 4 mmol) at room temperature. Methanol (50 mL) containing 2‐methylimidazole (3.2 g, 39 mmol) was subsequently added to the prior solution under stirring and refluxed at 60 °C for 30 min. After that, pink crystal precipitates were collected by centrifugation in fresh ethanol solution three times and vacuum dried at 60 °C overnight. The CoNC template was obtained by carbonization of the as‐prepared sample, the program was at 800 °C for 2 h, followed by 1100 °C for 1 h, and the rate was 30 °C min^−1^.

##### Synthesis of p‐FeTPPCl@CoNC Precursor

Tetraphenylporphyrin iron chloride (FeTPPCl, 50 mg, Sigma‐Aldrich), CoNC (100 mg), and chloroform anhydrous (20 mL) were added to a Schlenk flask and stirred for 3 h at room temperature. Anhydrous aluminum chloride (150 mg) was subsequently added into the above solution under stirring for 2 days at 65 °C. After that, the precipitate was collected by filtering and washed with methanol, dichloromethane, tetrahydrofuran, *N,N*‐dimethylformamide, and acetone, respectively. The powder was further purified by Soxhlet extractions for 24 h with methanol and dichloromethane, respectively. After drying in a vacuum oven at 60 °C for 12 h, the solid p‐FeTPPCl@CoNC was obtained as the precursor for subsequent processes.

##### Synthesis of p‐FeNC@CoNC

The as‐obtained p‐FeTPPCl@CoNC precursor was treated at 1100 °C for 1 h in an N_2_ atmosphere with a quick ramp rate of 30 °C min^−1^.

##### Characterization

Catalyst morphology was determined using SEM on a Hitachi SU 70 microscope at a working voltage of 5 kV. Aberration‐corrected HAADF and bright‐field (BF) STEM images and EELS were acquired on a Nion UltraSTEM in the Center for Nanophase Materials Sciences at Oak Ridge National Laboratory. The instrument was operated with an accelerating voltage of 60 kV and was equipped with a Gatan Enfina EELS spectrometer. XPS was conducted using a Kratos AXIS Ultra DLD XPS system equipped with a hemispherical energy analyzer and a monochromatic Al Kα source. The source was operated at 15 keV and 150 W; pass energy was fixed at 40 eV for the high‐resolution scans. Determination of catalyst surface area and porosity distribution was achieved using the BET analyses of the N_2_ isothermal sorption measurement recorded at 77 K on a Micrometritics TriStar II with samples’ degassing condition for 6 h at 150 °C under vacuum. Raman spectra of catalysts coated on glass slides were recorded with a wavelength of 514 nm laser at ambient conditions by the Renishaw Raman system. The Gaussian function was used for the fitting of the spectra. Fe and Co K‐edge XAS was measured at beamline 12BM, Advanced Photon Source (APS), Argonne National Laboratory (ANL). Data reduction, data analysis, and EXAFS fitting were conducted with the Athena, Artemis, and IFEFFIT software packages.

## Conflict of interest

The authors declare no conflict of interest.

## Data Availability Statement

The data that support the findings of this study are available from the corresponding author upon reasonable request.

## Supporting information

Supplementary Material
