# Supplementary material for: Binary Atomically Dispersed Metal‐Site Catalysts with Core−Shell Nanostructures for O2 and CO2 Reduction Reactions
Source: Small Sci. 2021 Aug 5;1(10):2100046. doi: 10.1002/smsc.202100046 (PMC11935902; doi:10.1002/smsc.202100046)
Supplement: Supplementary file 1 — Supplementary Material [file SMSC-1-2100046-s001.pdf]

Supporting Information

DOI: 10.1002/ ((please add manuscript number))
Article type: Research Paper

Binary Atomically Dispersed Metal Site Catalysts with Core-Shell Nanostructures for O_2 and CO_2 Reduction Reactions

*Xiaoxuan Yang, Maoyu Wang, Michael J. Zachman, Hua Zhou, Yanghua He, Shengwen Liu, Hong-Ying Zang**, Zhenxing Feng**, and Gang Wu**

X. Yang, Prof. H.-Y. Zang
Key Laboratory of Polyoxometetalate Science of the Ministry of Education,
Faculty of Chemistry,
Northeast Normal University,
Changchun, Jilin 130024, China
E-mail: zanghy100@nenu.edu.cn

X. Yang, Y. He, Dr. S. Liu, Prof. G. Wu
Department of Chemical and Biological Engineering,
University at Buffalo, The State University of New York,
Buffalo, NY 14260, United States
E-mail: gangwu@buffalo.edu

M. Wang, Prof. Z. Feng
School of Chemical, Biological, and Environmental Engineering,
Oregon State University,
Corvallis, OR 97331, United States
E-mail: zhenxing.feng@oregonstate.edu

Dr. M. J. Zachman
Center for Nanophase Materials Sciences,
Oak Ridge National Laboratory,
Oak Ridge, TN 37831, United States

Dr. H. Zhou
X-Ray Science Division,
Argonne National Laboratory,
Argonne, IL 60439, United States

Experimental sections

Synthesis of NiNC template: Typically, zinc (II) nitrate hexahydrate (6.78 g) were dissolved in 150 mL methanol in a round-bottom flask. The other 150 mL methanol solution containing 2-methylimidazole (7.88 g) was subsequently added to the above solution, shook vigorously, and placed in an oven at 60 ^oC for 24 hours. The nanocrystals were collected by centrifugation, washed for three times with ethanol, and then dried at 60 ^oC in a vacuum oven for overnight. The precursors were subsequently carbonized at 800 ^oC under N_2 flow for 2 hours followed by 1100 ^oC for 1 hour at a heating rate of 30 ^oC min^{-1}. The powder (50 mg) was dispersed in isopropanol (5 mL) containing nickle chloride (2.5 mg) followed by sonication for 1 hour and then stirring for 2 hours. After centrifugation and vacuum drying, thermal activation was performed at 1100 ^oC for 1 hour at a heating rate of 30 ^oC min^{-1}.

Synthesis of p-FeTPPCl@NiNC precursor and p-FeNC@NiNC: The p-FeTPPCl@NiNC was prepared following the same procedure of p-FeTPPCl@CoNC, where the CoNC was replaced by NiNC. The p-FeNC@NiNC was prepared following the same procedure of p-FeNC@CoNC.

Synthesis of FeNC template: Zinc nitrate hexahydrate (3.39 g) and iron nitrate nonahydrate (100 mg) were dissolved in 300 mL methanol in a round-bottom flask. Methanol (300 mL) containing 2-methylimidazole (3.94 g) was subsequently added to the prior solution under stirring, and then kept at 60 ^oC for 24 hours. The nanocrystals were collected by centrifugation, washed with ethanol for three times and vacuum dried at 60 ^oC for overnight. The FeNC template was obtained by carbonization of the as-prepared sample, the program was 800 ^oC for 2 hours followed by 1100 ^oC for 1 hour, and the rate was 30 ^oC min^{-1}.

Synthesis of p-FeTPPCl@FeNC precursor and p-FeNC@FeNC: The p-FeTPPCl@FeNC was prepared following the same procedure of p-FeTPPCl@CoNC, where the CoNC was replaced by FeNC. Also, the p-FeNC@FeNC was prepared following the same procedure of p-FeNC@CoNC.

Synthesis of NC template: The NC was prepared following the same procedure of FeNC template but without iron nitrate nonahydrate.

Synthesis of p-H_2TPP@NC precursor and p-NC@NC: The p-H_2TPP@NC was prepared following the same procedure of p-FeTPPCl@CoNC, where the FeTPPCl and CoNC template were replaced by tetraphenylporphyrin (H_2TPP) and NC template, respectively. Also, the p-NC@NC was prepared following the same procedure of p-FeNC@CoNC.

Electrochemical measurements

The ORR testing: Electrochemical measurements were performed using an electrochemical workstation (CHI760b) equipped with a rotating-ring disc electrode (RRDE, Pine, AFMSRCE 3005) in a conventional three-electrode cell. The RRDE with thin catalyst film, Hg/$HgSO_4$ (K_2SO_4-sat.) electrode, and graphite rod were used as the working, reference and counter electrodes, respectively. Before each measurement, the reference electrode was calibrated to a reversible hydrogen electrode (RHE) in the same electrolyte. To prepare the working electrode, 5 mg catalysts were ultrasonically dispersed in a 0.5 mL mixture of isopropyl alcohol and Nafion® (5 wt.%) solution for 30 min. The ink was subsequently drop-casted on the disk electrode with a

designed loading of 0.6 mg cm^{-2}, and dried at room temperature to yield a thin-film electrode. Cyclic voltammetry (CV) measurement was first used to activate the catalyst film in O_2-saturated 0.5 M H_2SO_4 at a scan rate of 50 mV s^{-1} and a rotation rate of 200 rpm. The electrocatalytic activity for the ORR was tested by steady-state measurement using potential staircase control with a step of 0.05 V at an interval of 30 s from 1.0 to 0 V *vs.* RHE with a rotation rate of 900 rpm in O_2-saturated 0.5 M H_2SO_4 solution at 25 ^{o}C. Catalyst stability was studied by potential cycling (0.6 to 1.0 V in O_2-saturated 0.5 M H_2SO_4, or 1.0 to 1.5 V in N_2-saturated 0.5 M H_2SO_4) at 25 ^{o}C or 60 ^{o}C, and by holding at the constant potential at 0.85 V or 0.7 V at 25 ^{o}C during the ORR. The hydrogen peroxide (H_2O_2) yield and electron transfer number (n) were calculated from the recorded ring (I_r) and disk current (I_d) using the following equations where N = 0.37 is collection efficiency:

$$n = 4 \times \frac{I_d}{I_d + I_r / N}$$

$$H_2O_2\% = 200 \times \frac{I_r / N}{I_d + I_r / N}$$

Fuel cell testing: The catalyst/ionomer ink was prepared by sonication of the mixture of catalyst (40 mg), Nafion® (5 wt.%) solution (480 mg), isopropanol (480 mg), and deionized water (480 mg) for 3 hours under ice-water bath. The cathode was obtained by repeatedly brushing the catalyst ink on the carbon paper (5 cm^2) until the loading of about 4.0 mg cm^{-2} was achieved. A commercial Pt-catalyzed gas diffusion electrode was used as the anode. The MEA was prepared by hot-pressing Nafion® 212 membrane coupled with anode and cathode at 130 ^{o}C for 5 min.

The MEA was measured by a fuel cell test station (Scribner 850e). Pure H_2 and air humidified at 80 ^{o}C were supplied to the anode and cathode, at a flow rate of 300 and 500 mL min^{-1}, respectively. Fuel cell polarization plots were recorded using standard fuel cell test stations (Fuel Cell Technologies Inc.) in a voltage control mode.

The CO_2RR testing: Electrochemical CO_2RR tests were carried out in a three-electrode H-cell in CO_2-saturated 0.5 M $KHCO_3$ electrolyte with a working electrode, a saturated Ag/AgCl reference electrode, and a platinum foil as a counter electrode. The catalyst ink was prepared by dispersing 3 mg catalysts in a mixture solution of DI-water (200 μL), ethanol (370 μL), and Nafion solution (5 wt.%, 30 μL) *via* sonication for 3 hours under ice-water bath. The ink was subsequently drop-casted onto a 1×0.5 cm^2 carbon paper with a designed loading of 0.4 mg cm^{-2}, and dried under an infrared lamp. The total volume of each compartment is 50.0 mL, with each compartment filled with 40.0 mL 0.5 M $KHCO_3$ and the two separated by a piece of Nafion® 115 membrane. Before each measurement, the high-purity CO_2 was introduced in the cathode chamber for 1 hour with a flow rate of 34 mL min^{-1}, and the catholyte was stirred at approximately 800 rpm throughout the test. CV was repeatedly performed until stable anodic and cathodic currents were observed. Linear sweep voltammetry (LSV) at a scan rate of 40 mV s^{-1} and chronoamperometric measurements were both executed using iR compensation for accounting for the solution resistance between the working and counter electrode. Also, the measured potentials were rescaled to the reversible hydrogen electrode by $E_{(RHE)} = E_{(Ag/AgCl)} + 0.199\ V + 0.0591V \times pH$.

Product Analysis: For the faradaic efficiency determination, 1 mL of the aliquot gas from the headspace of the cathode (after 10 minutes of chronoamperometry at select potentials) is injected into an online gas chromatography system (Agilent 7890B) equipped with two HP-Plot Q Capillary Columns and an HP-Plot Molesieve (Agilent) column, the thermal conductivity detector (TCD) for H_2 detection and a mechanized-assisted flame ionization detector (FID) for CO and CH_4 detection.

Relevant Calculations: Faradaic efficiency (FE) of evolved gases at each applied potential was calculated using the following equation:

$$FE = \frac{z \times P \times F \times V \times v_i}{R \times T \times j}$$

z = number of electrons transferred per mole of gas, which is 2 for H_2 and CO and 8 for CH_4;

P = atmospheric pressure (1.01×10^5 Pa);

F = Faraday's constant (96500 C mol^{-1});

V = the volume flow rate of the CO_2 supplied to the H-cell throughout electrolysis (30 mL min^{-1} or 5.0 m^3 s^{-1});

v_i = the concentration of gas products determined by GC (ppm);

R = the gas constant (8.314 J mol^{-1} K^{-1});

T = temperature (298.15 K);

j = current density from the potentiometric test at exactly 600 s.

The partial current density of CO, j_{CO} (or other evolved gases) can subsequently be calculated by

multiplying the obtained FE with the total current density.

TOF for CO production was calculated as follow[1]:

$$\text{TOF} = \frac{I_{product} / zF}{m_{cat.} \times \omega / M_{metal}} \times 3600$$

$I_{Product}$ = partial current for certain product, CO;

z = the number of electrons transferred for CO production, which is 2 for CO;

F = Faradaic constant, 96485 C mol^{-1};

$m_{cat.}$= the mass of catalyst on the electrode, g;

ω = metal loading in the catalyst based on XPS results;

M_{metal} = atomic mass of Ni (58.69 g mol^{-1}) for NiNC, atomic mass of Fe (55.85 g mol^{-1}) for p-FeNC, and atomic mass of 57.95 g mol^{-1} for p-FeNC@NiNC (based on the ratio of Ni and Fe).

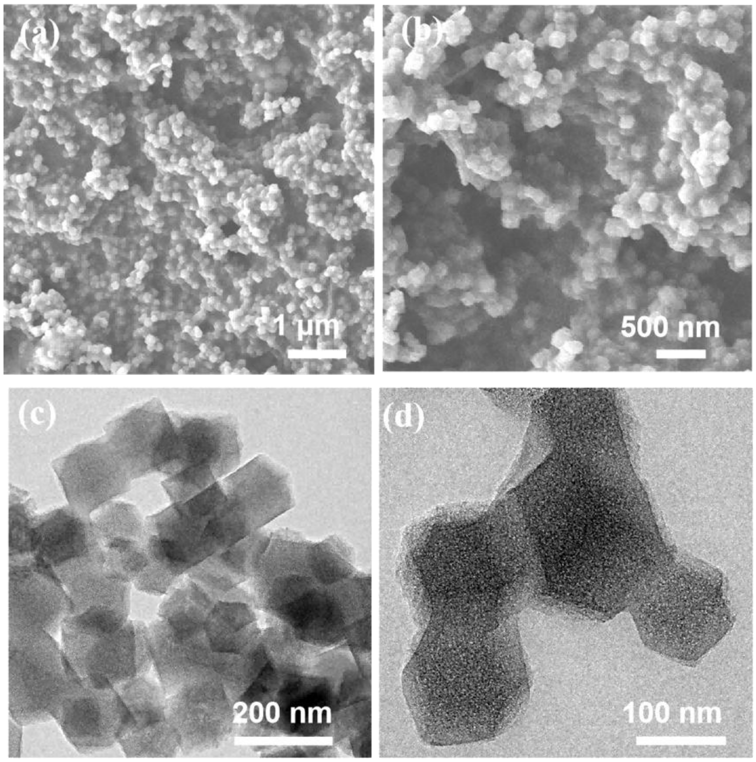

Figure S1. (a, b) SEM and (c, d) TEM images of the p-FeNC@CoNC catalyst.

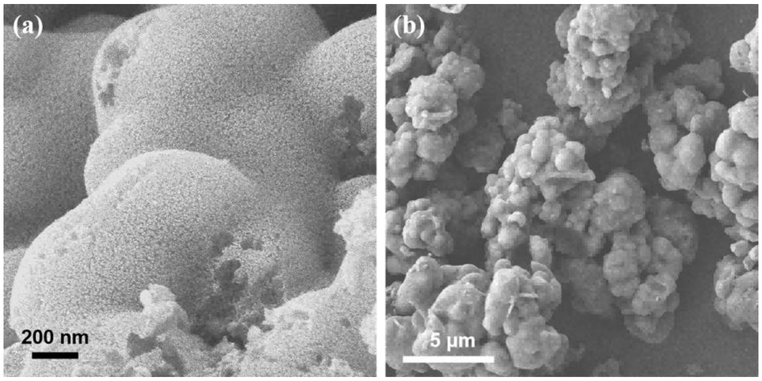

Figure S2. SEM images of the p-FeNC after heat treatment.

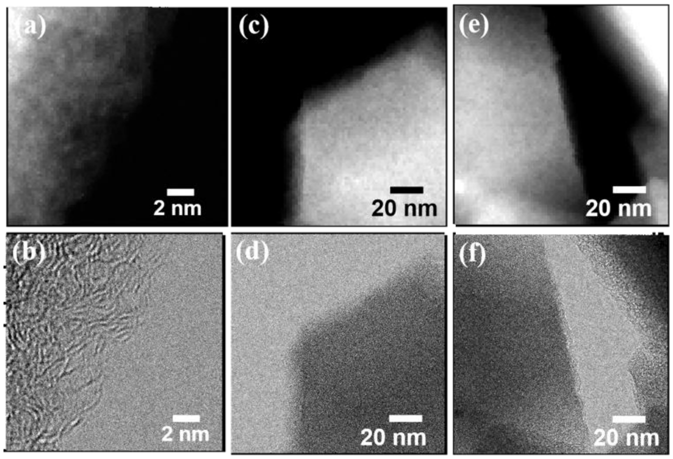

Figure S3. High-angle annular dark-field STEM (top) and bright-field STEM (bottom) images of the p-FeNC@CoNC catalysts showing their well-defined morphologies and sharp edges, as well as their crumpled graphitic carbon lattice structure.

Electron energy-loss spectroscopy: Bonding and antibonding molecular orbitals that are rotationally symmetric about the axis connecting the atoms are called σ and σ* states, respectively. Non-rotationally symmetric bonding and antibonding orbitals are denoted π and π*. Various carbon allotropes such as diamond, graphite, and amorphous carbon have different carbon-carbon bonds.[2] For example, diamond contains only σ and σ* bonds, while graphite and amorphous carbon contain different combinations of σ/σ* and π/π* bonds, as depicted in **Figure 2**. The carbon K-edge is sensitive to the occupation of the antibonding orbitals and electron energy-loss spectroscopy (EELS) can therefore be used to identify different bonding states of carbonaceous materials. **Figure 2** in the main text utilizes this ability to demonstrate that the p-FeNC@CoNC catalysts are composed primarily of graphitic carbon.

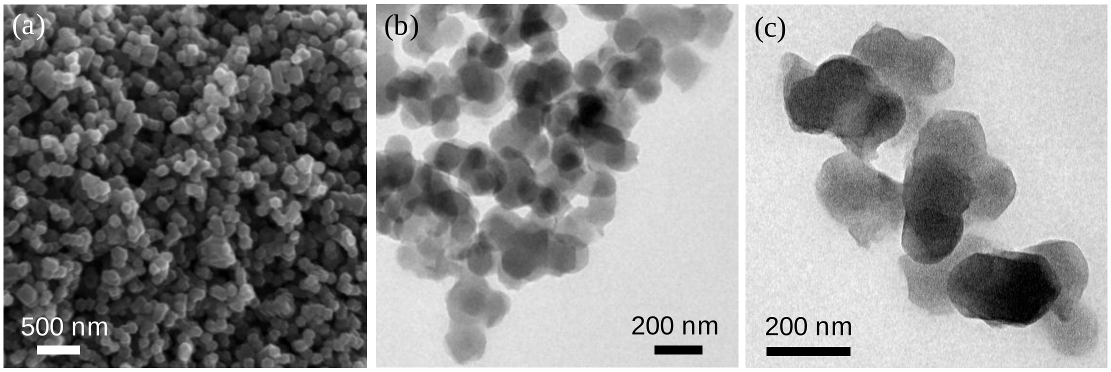

Figure S4. (a) SEM and (b, c) TEM images of the p-FeNC@NiNC sample.

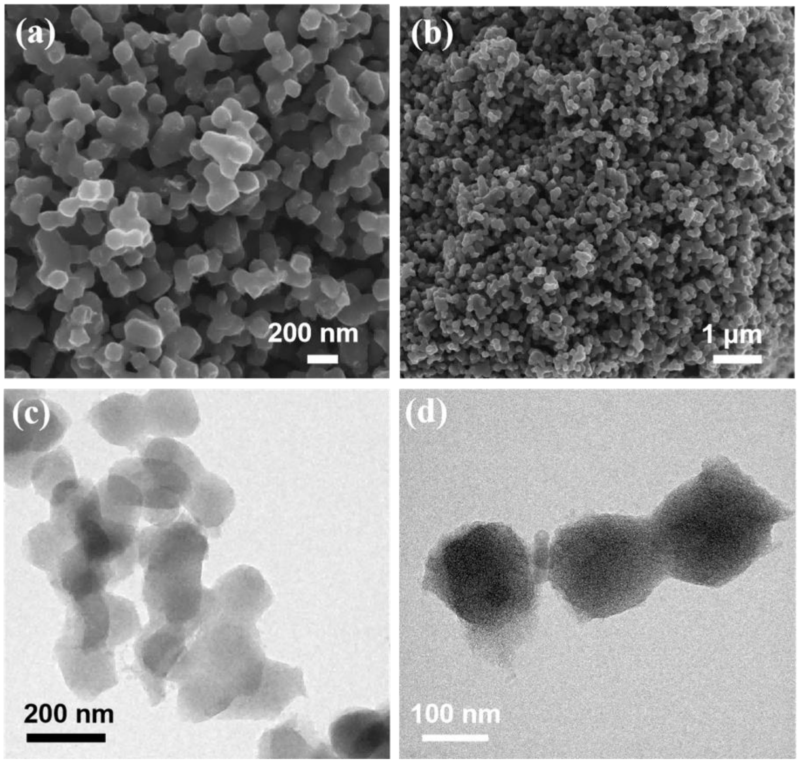

Figure S5. (a, b) SEM and (c, d) TEM images of the p-FeNC@FeNC sample with different magnifications.

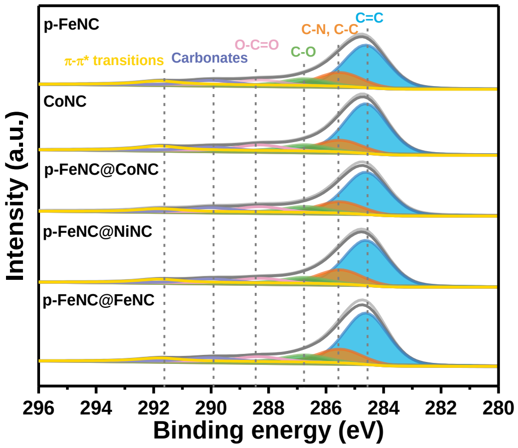

Figure S6. XPS C 1s analysis of different catalysts.

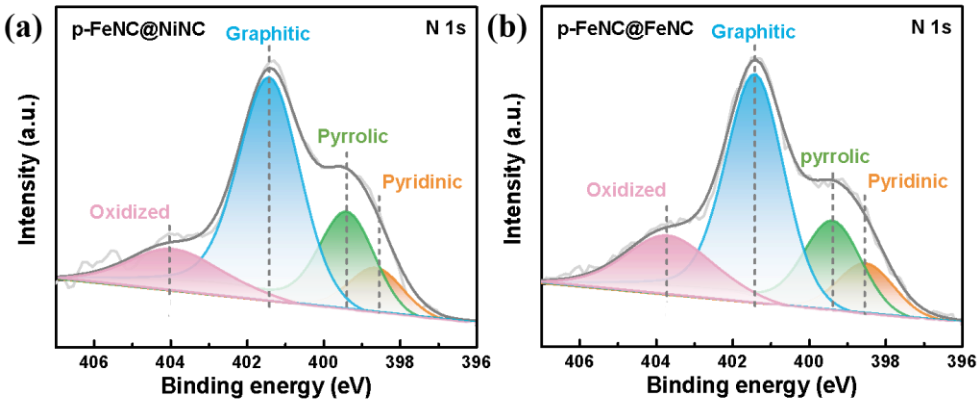

Figure S7. XPS N 1s analysis of (a) p-FeNC@NiNC and (b) p-FeNC@FeNC samples.

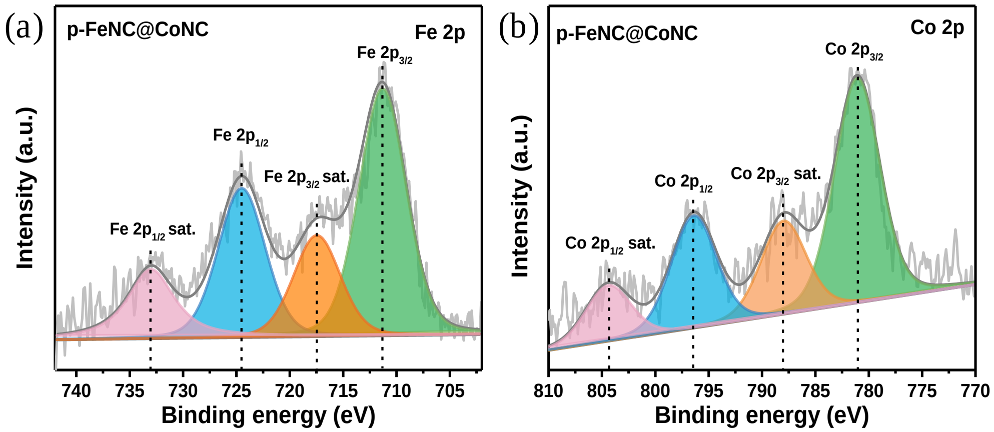

Figure S8. XPS characterizations of p-FeNC@CoNC. (a) Co 2p spectrum and (b) Fe 2p spectrum.

The Co 2p XPS peak is composed of the Co $2p_{1/2}$ sat., Co $2p_{1/2}$, Co $2p_{3/2}$ sat., and Co $2p_{3/2}$ peaks, which are located at 804.0, 796.4, 786.8, and 781.2 eV, respectively.[3] The XPS peaks of the Co $2p_{1/2}$ (Co $2p_{3/2}$) are exactly located between the Co^{2+} $2p_{1/2}$ (Co^{2+} $2p_{3/2}$) and Co^0 $2p_{1/2}$ (Co^0 $2p_{3/2}$), demonstrating that the single Co sites are positively charged due to the coordinatization of pyridinic N, and the valence is situated between +2 and 0.[4] Similarly, through the high-resolution Fe 2p XPS spectrum, the main peaks can be deconvoluted, and the valence state of Fe atoms can be determined at +2 to 0, inferring that the isolated diatomic Co-Fe metal–nitrogen sites are formed in the p-FeNC@CoNC sample.

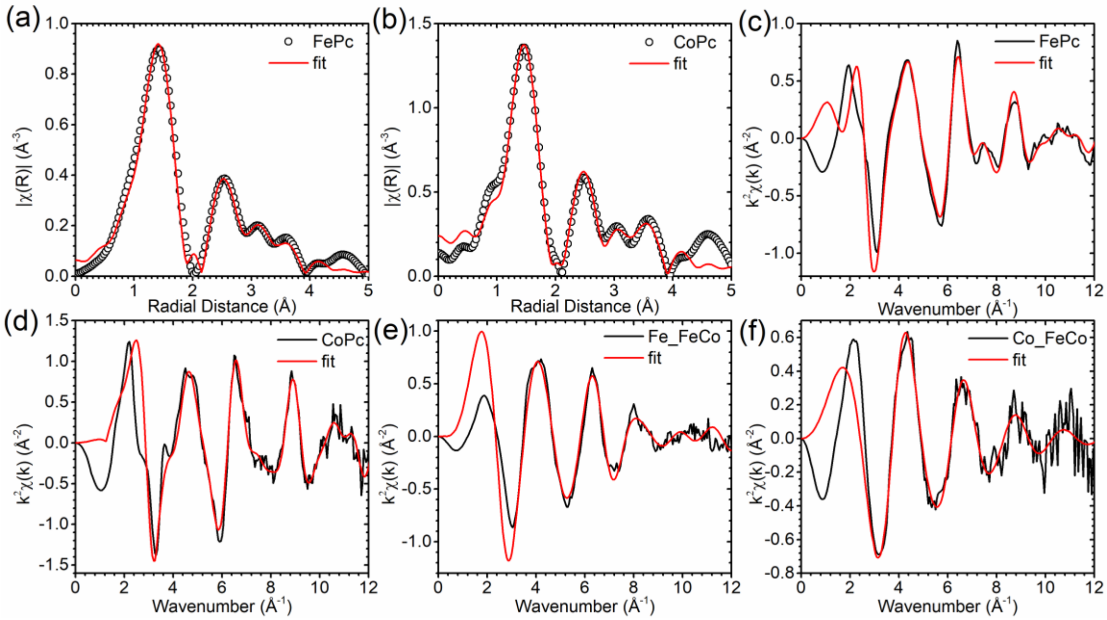

Figure S9. Fourier Transfer R-space EXAFS fitting for (a) Fe K-edge FePc and (b) Co K-edge CoPc. Fourier Transfer k-space EXAFS fitting for (c) Fe K-edge FePc, (d) Co K-edge CoPc, (e) Fe K-edge FeCo, and (f) Co K-edge FeCo.

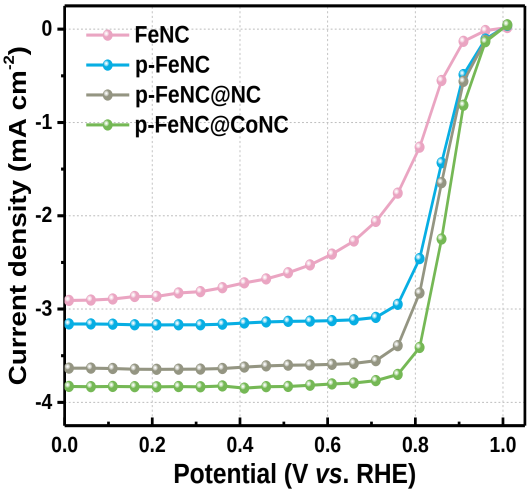

Figure S10. Steady-state ORR polarization plots of other reference catalysts in 0.5 M H_2SO_4 at 25 ^oC.

Relative to FeTPPCl-derived FeNC catalyst, p-FeNC catalyst derived from polymerized FeTPPCl could avoid metal agglomeration, which would be favorable for the formation of Fe-N_4 active sites as much as possible, and the introduction of NC could further expose more accessible active sites. Despite the insignificant enhancement on the ORR performance, the introduction of Co-N_4 sites could remarkably enhance the catalytic durability, which was further verified under various accelerated stability tests in the subsequent experimental observation.

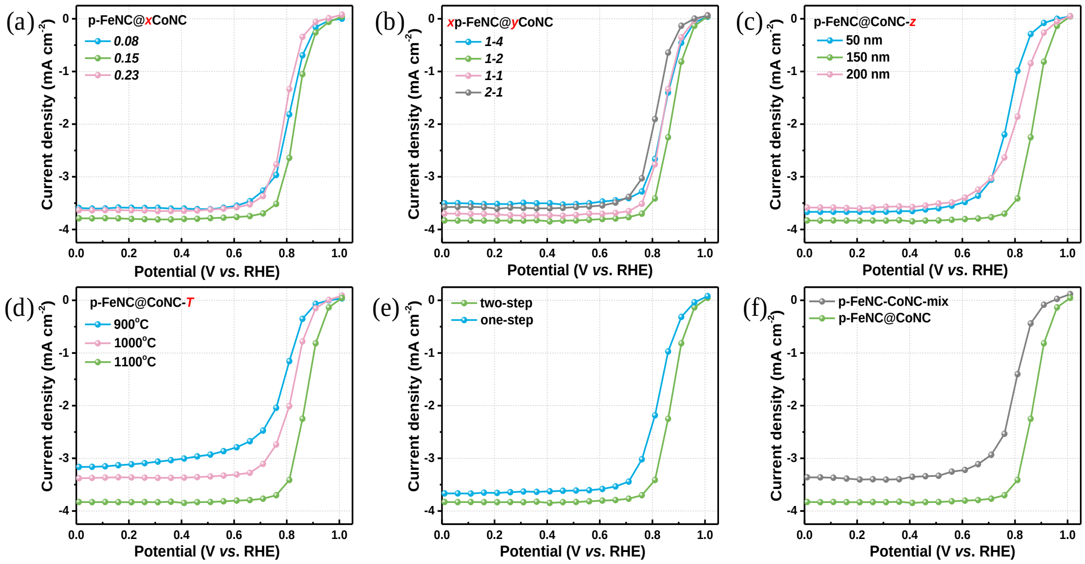

Figure S11. Different effects on the ORR activity of the p-FeNC@CoNC catalyst. (a) The Co content, (b) mass ratios of shell to core, (c) sizes of Co-doped ZIF-8 precursor, (d) carbonization temperatures, (e) carbonization procedures of Co-doped ZIF-8 precursor, and (f) with and without core-shell structure formed.

The effect of Co doping content on ORR activity followed a “volcano plot” trend (Figure S11a). For the p-FeNC@*x*CoNC catalyst, where *x* represents the proportion of Co^{2+} in the total moles of Co^{2+} and Zn^{2+} in the Co-ZIF-8. The activity was continuously increased as Co doping increased up to 0.15 and then decreased with higher Co doping. This experimental observation also verified the role of CoN_4 active sites in improving ORR activity to a certain extent, even though this effect was not as obvious as FeN_4 sites. Lower doping produces insufficient active site, but higher doping results in Co agglomeration and unfavorable carbon structures (*i.e.*, fewer defects

and porosities). A catalyst with an optimal balance between FeN_4 and CoN_4 is more desirable for conveying an excellent ORR activity by taking full advantage of each active site *via* a suitable ratio of shell-to-core (Figure S11b). The ORR activity is greatly dependent on the size of the particles (Figure S11c). The p-FeNC@CoNC with 150 nm delivered the most superior performance, hosting maximum number of exposed active sites, however, reducing the particle size to 50 nm leads to decreased ORR activity, due to significant particles agglomeration. Similarly, a higher temperature (up to 1100°C) leads to enhanced activity, which likely facilitates the formation of a larger number of active sites or sites with higher intrinsic activity (Figure S11d). The two-step carbonization on the CoZIF precursor strategy is very critical (Figure S11e). Compared with one-step strategy, the two-step thermal activation can generate relatively high activity. The first step at a relatively low temperature is to create more defects through the evaporation of Zn from the CoZIF precursor, and the second step at a higher temperature can generate active sites. Also, the sample prepared by manually grinding p-FeNC and CoNC (namely, p-FeNC-CoNC-mix) *via* the mechanical mixing method exhibits poor ORR performance (Figure S11f). It was characterized by SEM showing a chaotic structure, in which large agglomerated p-FeNC could be seen, and CoNC gathered on its surface without the formation of a core-shell structure, which was further implied that the tailored core-shell structure is effective in boosting accessible active sites for the catalytic activity (Figure S12).

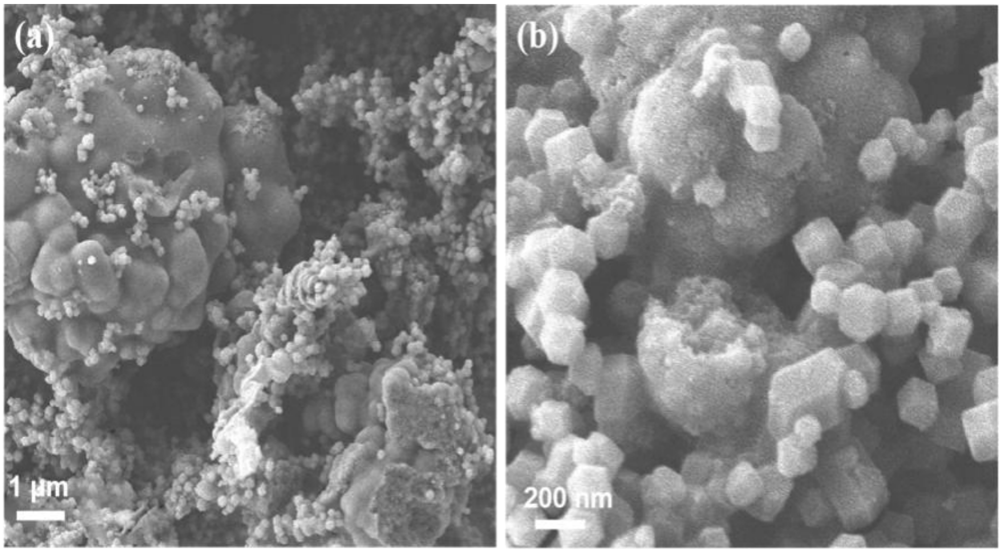

Figure S12. SEM images of p-FeNC-CoNC-mix sample.

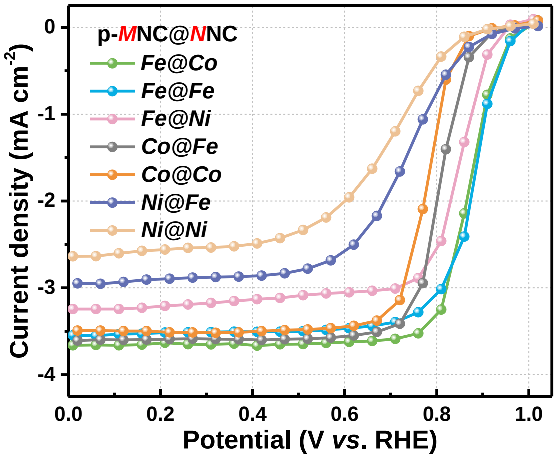

Figure S13. Steady-state ORR polarization plots of shell@core structured catalysts with different metal elements.

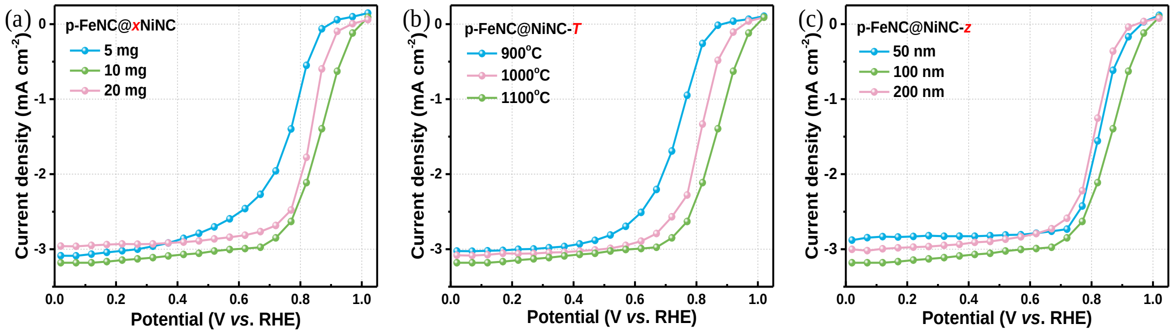

Figure S14. Different effects on the ORR activity of the p-FeNC@NiNC catalyst. (a) The Ni content, (b) the heating temperatures, and (c) the sizes of Ni-doped ZIF-8 precursor.

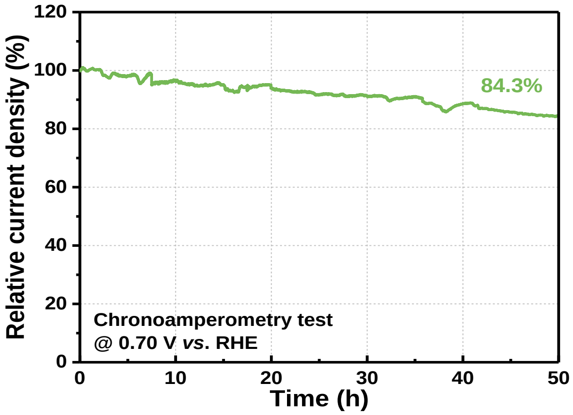

Figure S15. A 50-hour chronoamperometry test at constant potentials of 0.7 V *vs*. RHE for the p-FeNC@CoNC catalyst.

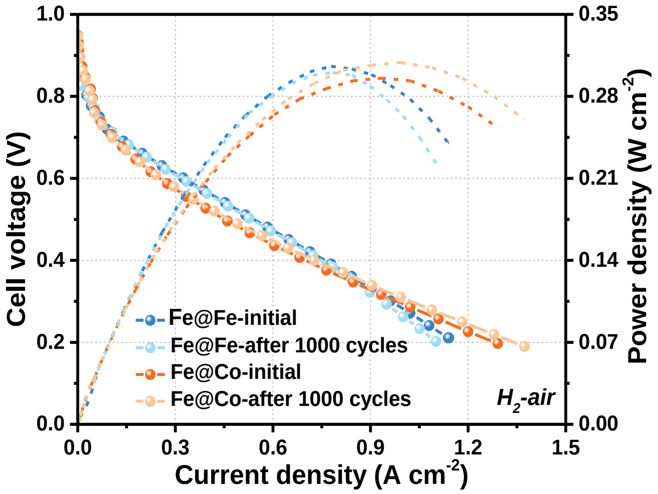

Figure S16. H_2-air fuel cell performance of the p-FeNC@CoNC catalyst, and p-FeNC@FeNC catalyst, respectively. Test conditions: anode: 0.2 mg_{Pt} cm^2 Pt/C; H_2 flow rate 200 sccm, 1.0 bar H_2 partial pressure; cathode: *ca*. 4.0 mg cm^2, 200 sccm gas flow rate, 1.0 bar total partial pressure of gas flow; membrane: Nafion® 211; cell: 80 °C, 100% RH, 5.0 cm^2 MEA electrode area.

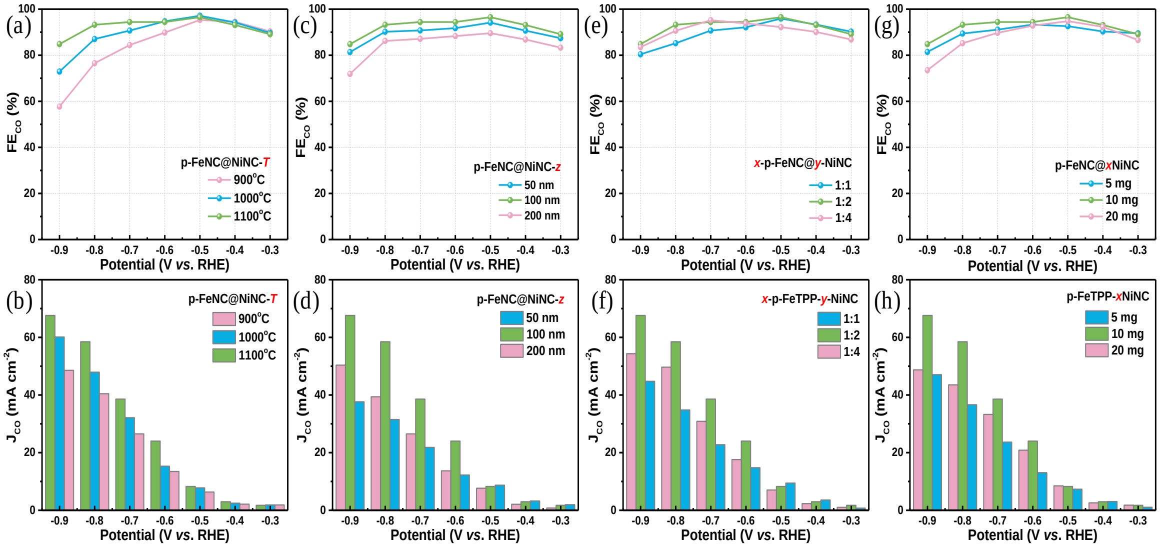

Figure S17. Different effects on the CO_2RR activity of the p-FeNC@NiNC catalyst. (a, b) Heating temperatures, (c, d) sizes of Ni-doped ZIF-8, (e, f) ratios of the shell to the core, and (g, h) Ni loading content.

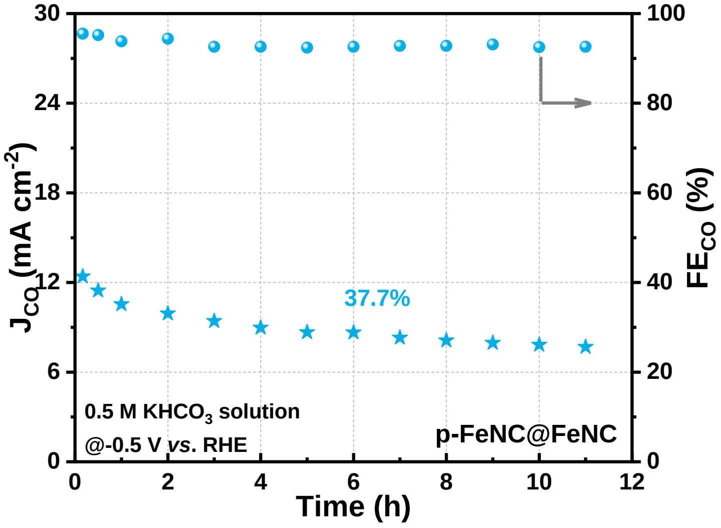

Figure S18. Chronoamperograms of the p-FeNC@FeNC at -0.5 V *vs*. RHE for 12 hours.

Table S1. Elemental quantification determined by XPS for different materials (at.%).

**Sample**	**C**	**N**	**O**	**Fe**	**Co**	**Ni**	**Zn**
**p-FeNC**	93.18	3.34	2.75	0.18	/	/	0.55
**CoNC**	92.17	3.55	3.50	/	0.17	/	0.61
**p-FeNC@CoNC**	90.57	2.33	6.59	0.13	0.11	/	0.27
**p-FeNC@NiNC**	90.44	2.35	6.63	0.14	/	0.12	0.32
**p-FeNC@FeNC**	90.68	2.23	6.54	0.22	/	/	0.33

Table S2. Fitting results for C 1s XPS spectra for different samples (at.%).

Sample	C=C	C-N, C-C	C-O	O-C=O	Carbonates	π-π*
**p-FeNC**	55.6	18.1	8.9	6.0	5.1	6.3
**CoNC**	60.8	14.4	7.8	6.9	3.7	6.4
**p-FeNC@CoNC**	59.2	16.1	8.2	6.7	5.0	4.8
**p-FeNC@NiNC**	59.7	16.6	7.7	6.4	4.3	5.3
**p-FeNC@FeNC**	60.4	16.1	8.3	6.2	4.3	4.7

Table S3. Fitting results for N 1s XPS spectra for different materials (at.%).

Sample	Pyridinic-N	Pyrrolic-N	Graphitic-N	Oxidized N
**FeNC**	20.0	23.9	42.6	13.5
**CoNC**	25.9	19.1	43.1	11.9
**p-FeNC@CoNC**	13.0	21.6	51.4	14.0
**p-FeNC@NiNC**	11.4	21.8	54.5	12.3
**p-FeNC@FeNC**	12.4	19.7	50.2	17.7

Table S4. Fitting parameters of the EXAFS spectra of standard FePc and CoPc, and sample p-FeNC@CoNC (denoted as FeCo) at Fe K-edge and Co K-edge (CN: coordination number; R: distance; E_0: energy shift; σ^2: mean-square disorder, R-factor: the goodness of fitting). The numbers in the parentheses are the last digit error. The numbers in the parentheses with yellow label are the full error.

Sample	Scattering Path	CN	R(Å)	E_0(eV)	σ^2(Å^2)	R-factor
**FePc**	Fe-N	4	1.93(1)	-5.2(1)	0.0078(9)	0.018
	Fe-C	8	2.96(1)		0.0078(1)	
	Fe-N-C	16	3.13(2)		0.0040(6)	
	Fe-N	4	3.37(2)		0.0065(8)	
	Fe-N-N	16	3.86(2)		0.0008(7)	
	Fe-N-N	4	3.86(2)		0.0008(7)	
	Fe-C	6	4.19(2)		0.0184(6)	
	Fe-N-C	12	4.22(2)		0.0107(2)	
**Fe in FeCo**	Fe-N	1.9(3)	1.94(3)	0.5(3)	0.0008(2)	0.017
	Fe-N	2.5(5)	2.09(3)		0.0008(2)	
	Fe-C	8.7(1.2)	2.97(4)		0.0156(1)	
	Fe-N-C	17.4(2.5)	3.12(7)		0.0205(1)	
**CoPC**	Co-N	4	1.92(1)	5.4(9)	0.0024(7)	0.012
	Co-C	8	2.94(1)		0.0027(3)	
	Co-N-C	16	3.11(1)		0.0187(1)	
	Co-N	4	3.35(1)		0.0094(5)	

	Co-N	4	3.84(1)		0.0008(7)	
	Co-C	6	4.17(1)		0.0200(7)	
	Co-N-C	12	4.19(1)		0.0041(7)	
	Co-N-C-N	6	4.22(1)		0.0008(7)	
	Co-N	4.3(7)	1.90(1)		0.0097(1)	
**Co in FeCo**	Co-C	8.7(1.3)	2.92(2)	-5.8(8)	0.0246(6)	0.012
	Co-N-C	17.3(2.7)	3.09(2)		0.0139(3)	

Table S5. Comparison of ORR performance for different nonprecious catalysts in acidic media.

**Catalyst**	$E_{1/2}$ **(V *vs*. RHE)**	**Electrolyte**	**Reference**
**p-FeNC@CoNC**	**0.87**		
**p-FeNC@NiNC**	**0.84**	**0.5 M H_2SO_4**	**this work**
**p-FeNC@FeNC**	**0.87**		
Fe-PANI/BP2000/NH_4Cl	0.80	0.1 M $HClO_4$	Angew. Chem. Int. Ed., 2020, 59 (4), 1627.
$FeCl_2$-NC-1000	0.80	0.1 M $HClO_4$	J. Am. Chem. Soc., 2020, 142 (3), 1417.
FeN_4/HOPC-c-1000	0.80	0.5 M H_2SO_4	Angew. Chem. Int. Ed., 2020, 59, 2688.
Fe-N_4/C-60	0.8	0.1 M $HClO_4$	Adv. Mater., 2020, 32, 2000966.
SnNC	0.74	0.1 M $HClO_4$; 1600 rpm	Nat. Mater., 2020, 19, 1215.
Fe-N/P-C-700	0.72	0.1 M $HClO_4$	J. Am. Chem. Soc., 2020, 142, 2404.
S-Cu-ISA/SNC	0.74	0.5 M H_2SO_4	Nat. Commun., 2020, 11, 3049.
1.5Fe-ZIF	0.88	0.5 M H_2SO_4	Energy Environ. Sci., 2019, 12, 2548.
Fe_2-N-C	0.78	0.5 M H_2SO_4	Chem, 2019, 5 (11), 2865.
FeNC-S-MSUFC-2	0.75	0.5 M H_2SO_4	J. Am. Chem. Soc., 2019, 141 (15), 6254.
Fe_2-Z8-C	0.805	0.5 M H_2SO_4	Angew. Chem. Int. Ed., 2018, 57, 1204.
p-Fe-N-CNFs	0.74	0.1 M $HClO_4$	Energy Environ. Sci., 2018, 11,

			2208.
20Co-NC-1100	0.8	0.5 M H_2SO_4	Adv. Mater., 2018, 30, 1706758.
SA-Fe-HPC	0.81	0.1 M H_2SO_4	Angew. Chem. Int. Ed., 2018, 57, 9038.

Table S6. Comparison of CO_2RR performance for different nonprecious catalysts.

Catalyst	FE_{CO}	J_{CO}	TOF	Stability	Reference
**p-FeNC@NiNC**	**97% (-0.5 V)**	**58.5 mA cm^{-2} (-0.8 V)**	**12058 h^{-1}**	**40 h**	**this work**
Co-N-Ni/NPCNSs	96.4% (-0.48 V)	12.9 mA cm^{-2} (-0.74 V)	2049 h^{-1}	20 h	Energy Environ. Sci., 2021, 14, 3019-3028
Ni-N_3-C	95.6% (-0.65 V)	6.64 mA cm^{-2} (-0.65 V)	1425 h^{-1}	10 h	Angew. Chem. Int. Ed. 2021, 60, 7607
Ni_1-N-C	96.8% (-0.8 V)	27 mA cm^{-2} (-0.8 V)	11315 h^{-1}	10 h	Angew. Chem. Int. Ed., 2020, 59, 20589-20595
NiSA-N_2-C	98% (-0.8 V)	/	~3467 h^{-1}	10 h	Angew. Chem. Int. Ed., 2020, 59, 2705-2709
Ni/Fe-N-C	98% (-0.7 V)	19.7 mA cm^{-2} (-1.0 V)	7682 h^{-1}	30 h	Angew.Chem.Int. Ed. 2019, 58,6972-6976
NiPor-CTF	97% (-0.9 V)	52.9 mA cm^{-2} (-0.9 V)	1701 h^{-1}	20 h	Adv. Funct. Mater. 2019, 1806884.
Ni-N-MEGO	92% (-0.7 V)	26.8 mA cm^{-2} (-0.7 V)	864 h^{-1}	20 h	Appl. Catal. B-Environ. 2019, 243, 294.
FePGH	96% (-0.39 V)	9.67 A mg^{-1} (-0.69 V)	9360 h^{-1}	20 h	Energy Environ. Sci. 2019, 12, 747.
Co-N_4	82% (-0.8 V)	15.8 mA cm^{-2} (-1.0 V)	1455 h^{-1}	10 h	Appl. Catal. B-Environ. 2019, 240, 234.
CoPP@CNT	98% (-0.6 V)	/	7560 h^{-1}	12 h	Angew. Chem. Int. Ed. 2019, 58, 6595.
ZnS	94% (-0.8 V)	~11 mA cm^{-2} (-1.0 V)	1500 h^{-1}	15 h	J. Mater. Chem. A 2019, 7, 1418.
Cu-APC	92% (-0.78 V)	8.6 mA cm^{-2} (-0.78 V)	/	3 h	Nat. Chem. 2019, 11, 222.
Ni-CNT-CC	99% (-0.83 V)	32.3 mA cm^{-2} (-0.83 V)	100179 h^{-1}	100 h	Angew. Chem. Int. Ed., 2019, 59, 798-803
Ni-N_4 SAC	90% (-0.9 V)	65 mA cm^{-2} (-0.9 V)	135000 h^{-1}	14 h	Angew. Chem. Int. Ed., 2019, 59, 1961-1965.
C-Zn_1Ni_4	98% (-0.83 V)	71.5 ± 2.9 mA cm^{-2} (-1.03 V)	10087 h^{-1}	12 h	Energy Environ. Sci. 2018, 11, 1204.
Co-N_5	99% (-0.73 V)	4.5 mA cm^{-2} (-0.73 V)	480.2 h^{-1}	10 h	J. Am. Chem. Soc. 2018, 140, 4218.

References

[1] Yan, C.; Li, H.; Ye, Y.; Wu, H.; Cai, F.; Si, R.; Xiao, J.; Miao, S.; Xie, S.; Yang, F.; Li, Y.; Wang, G.; Bao, X., *Energy & Environmental Science* **2018,** *11* (5), 1204-1210.

[2] Garvie, L. A. J.; Craven, A. J.; Brydson, R., *American Mineralogist* **1994,** *79* (5-6), 411-425.

[3] Yin, G.; Yuan, X.; Du, X.; Zhao, W.; Bi, Q.; Huang, F., *Chemistry – A European Journal*

2018, *24* (9), 2157-2163.

[4] Yin, P.; Yao, T.; Wu, Y.; Zheng, L.; Lin, Y.; Liu, W.; Ju, H.; Zhu, J.; Hong, X.; Deng, Z.; Zhou, G.; Wei, S.; Li, Y., *Angewandte Chemie International Edition* **2016,** *55* (36), 10800-10805.
